# Mutants of phage bIL67 RuvC with enhanced Holliday junction binding selectivity and resolution symmetry

**DOI:** 10.1111/mmi.12343

**Published:** 2013-08-14

**Authors:** Victoria Green, Fiona A Curtis, Svetlana Sedelnikova, John B Rafferty, Gary J Sharples

**Affiliations:** 1 Krebs Institute, Department of Molecular Biology and Biotechnology, University of SheffieldWestern Bank, Sheffield, S10 2TN, UK; 2 School of Biological and Biomedical Sciences, Biophysical Sciences Institute, Department of Chemistry, Durham UniversityScience Site, Durham, DH1 3LE, UK

## Abstract

Viral and bacterial Holliday junction resolvases differ in specificity with the former typically being more promiscuous, acting on a variety of branched DNA substrates, while the latter exclusively targets Holliday junctions. We have determined the crystal structure of a RuvC resolvase from bacteriophage bIL67 to help identify features responsible for DNA branch discrimination. Comparisons between phage and bacterial RuvC structures revealed significant differences in the number and position of positively-charged residues in the outer sides of the junction binding cleft. Substitutions were generated in phage RuvC residues implicated in branch recognition and six were found to confer defects in Holliday junction and replication fork cleavage *in vivo*. Two mutants, R121A and R124A that flank the DNA binding site were purified and exhibited reduced *in vitro* binding to fork and linear duplex substrates relative to the wild-type, while retaining the ability to bind X junctions. Crucially, these two variants cleaved Holliday junctions with enhanced specificity and symmetry, a feature more akin to cellular RuvC resolvases. Thus, additional positive charges in the phage RuvC binding site apparently stabilize productive interactions with branched structures other than the canonical Holliday junction, a feature advantageous for viral DNA processing but deleterious for their cellular counterparts.

## Introduction

Resolution of Holliday structure joint molecules either completes genetic recombination by severing the strand links between coupled chromosomes or triggers fresh exchanges at regressed replication forks (Atkinson and McGlynn, 2009). A structurally diverse family of endonucleases is responsible for DNA junction processing, many of which have conspicuous preferences in the branched structures they recognize and nucleotide sequences they cleave. Resolving enzymes are classically homodimeric, metal ion-dependent endonucleases with a marked preference for binding junction DNA. Each of the known resolution endonuclease family subgroups differs in the way its members recognize and cleave branched junctions. There are eight distinct classes: T7 endonuclease I (Hadden *et al*., 2007), T4 endonuclease VII (Biertumpfel *et al*., 2007), RusA (Rafferty *et al*., 2003), Rap (Sharples *et al*., 2004), RuvC/Cce1/Ydc2/A22R (Ariyoshi *et al*., 1994; Garcia *et al*., 2000; Ceschini *et al*., 2001), RecU (Ayora *et al*., 2004; McGregor *et al*., 2005), Hjc/Hje (Bond *et al*., 2001; Nishino *et al*., 2001; Middleton *et al*., 2004) and Yen1/GEN1 (Ip *et al*., 2008). Cellular Holliday junction resolvases need to be highly selective in the structures they cleave as unwarranted breaks at forks or bubbles in DNA may prove disastrous for replicating cells. This does not hold true for viral resolution endonucleases, which are generally less discriminating than their cellular counterparts, targeting a range of branched DNA forms in addition to Holliday structures (Culyba *et al*., 2007; Declais and Lilley, 2008). This appears to be an evolutionary adaptation to process any branched intermediates that may potentially interfere with virion assembly.

In bacteria, two alternate Holliday junction resolvases, RuvC in Gram-negatives and RecU in Gram-positives, function in a tripartite complex with the RuvAB branch migration machinery (Sharples *et al*., 1999; Yamada *et al*., 2004). Hence, mutation of any of the three subunits confers a similar DNA repair defect consistent with an inability to resolve intermediates of genetic recombination (Sharples *et al*., 1990; Sanchez *et al*., 2005). RuvC belongs to the RNase H/integrase superfamily of nucleases, including related resolving enzymes from yeast (Cce1/Ydc2) and poxviruses (A22R), and exhibits a high degree of selectivity for binding and cleaving X-shaped Holliday structures *in vitro* (Dunderdale *et al*., 1991; Garcia *et al*., 2000; Ceschini *et al*., 2001). This intrinsic specificity of resolution is achieved, in part, by a preference for bilateral strand incision at 5′-^A^/_T_TT↓^G^/_C_-3′ nucleotide sequences, located in opposing strands at the junction branch point (Shah *et al*., 1994). The two nicks are apparently made simultaneously, although in fact a single strand is cleaved initially, which in turn stimulates rapid cleavage of its partner within the lifetime of the junction–enzyme complex (Fogg and Lilley, 2000; Osman *et al*., 2009).

A distinct subset of the RuvC family (DUF3882 in the Conserved Domain Database; Marchler-Bauer *et al*., 2011) is found among selected Siphoviridae and Myoviridae phages isolated from Gram-positive lactococci and streptococci, despite the presence of the alternate RecU resolvase in these bacteria (Bidnenko *et al*., 1998; Curtis *et al*., 2005). These phage RuvC proteins are distantly-related to the bacterial RuvC resolving enzymes, although they share conserved acidic residues known to be critical for *Escherichia coli* RuvC (*Ec*RuvC) catalysis (Saito *et al*., 1995; Bidnenko *et al*., 1998). The RuvC from *Lactococcus lactis* phage bIL67 (67RuvC) was confirmed as a functional Holliday junction resolvase in both *in vitro* and *in vivo* experiments (Curtis *et al*., 2005). However, unlike *Ec*RuvC, it showed a relaxed structure-specificity, binding and cutting fork and X junctions almost equally well. It also differed in sequence-specificity, cleaving Holliday junctions preferentially at a reduced 5′-T↓^A^/_G_-3′ consensus (Curtis *et al*., 2005). Cleavage of fork structures occurred at the branch point in a largely sequence-independent manner and high-level expression of 67RuvC, and of a related enzyme from phage bIL66, induced the formation of chromosomal breaks by nicking at replication forks. Indeed plasmids expressing these phage resolving enzymes could not be recovered in strains lacking double-strand break repair pathways (Bidnenko *et al*., 1998; Curtis *et al*., 2005).

Precisely how Holliday junction resolvases distinguish between analogous branched structures and cleave preferentially at specific nucleotide sequences is not clear, partly due to the difficulty in obtaining crystal structures of resolving enzymes bound to appropriate DNA substrates; to date, only phage T4 and T7 resolvase structures have been obtained in complex with model X junctions (Biertumpfel *et al*., 2007; Hadden *et al*., 2007). The 67RuvC endonuclease provides an ideal opportunity to study how DNA structure and sequence selectivity are achieved by RuvC family resolving enzymes. To this end, we describe the crystal structure of a resolution-defective variant of 67RuvC and compare its structure with related bacterial RuvC and mitochondrial Ydc2 proteins. We also screened a number of conserved polar residues in 67RuvC by site-directed mutagenesis, taking advantage of rapid *in vivo* experiments to monitor replication fork cleavage and Holliday junction resolution. Two of the mutants identified have been characterized further, providing insight into how junction recognition within the DNA binding cleft differs between phage and bacterial RuvC endonucleases. Remarkably, these single amino acid substitutions have a dramatic effect on DNA selectivity and the symmetry of Holliday junction resolution, converting the phage endonuclease into an enzyme that resembles much more closely its cellular counterparts.

## Results

### Atomic structure of bIL67 RuvC D8N

The lactococcal phage bIL67 RuvC endonuclease is active on a range of branched DNA structures, including Holliday junctions and forks both *in vitro* and *in vivo* (Curtis *et al*., 2005). In contrast, its orthologue from *E. coli* resolves 4-stranded X junctions exclusively, ensuring that no inadvertent chromosomal breaks are introduced by nicking at replication forks (Dunderdale *et al*., 1991; Garcia *et al*., 2000; Ceschini *et al*., 2001). To investigate the modifications responsible for this difference in structure selectivity, the crystal structure of a 67RuvC D8N mutant was determined at 1.8 Å resolution. This nuclease deficient mutant corresponds to an *Ec*RuvC D7N mutant, which lacks one of four essential catalytic carboxylates, and was chosen to facilitate co-crystallization efforts in the presence of branched and unbranched DNA substrates. The lack of fork nicking activity fortuitously improves protein yield, simplifying the purification protocol (Curtis *et al*., 2005).

Purified 67RuvC D8N was screened for crystallization and well-diffracting crystals obtained in a P-orthorhombic space group subsequently confirmed as P2_1_2_1_2_1_. Following unsuccessful efforts to determine the structure of 67RuvC by molecular replacement utilizing models derived from *Ec*RuvC (Ariyoshi *et al*., 1994; Chen *et al*., 2013), the structure was solved using phases determined from a selenomethionine-incorporated form of the protein with data collected in a multiwavelength anomalous dispersion (MAD) experiment at three wavelengths (Table S1). The structural model was refined against a 1.7 Å data set collected at the high energy remote wavelength. Later trials involving a high concentration of Mg^2+^ produced crystals which diffracted to 1.8 Å. A data set was collected and the structure of 67RuvC with Mg^2+^ bound was determined by molecular replacement using the earlier structure as a search model. Data statistics and analysis of model quality of both the apo and metal-bound forms are presented in Table 1 and an example of the final σA-weighted 2mFo-DFc electron density map is shown in Fig. S1.

**Table 1:** Data processing and structure refinement statistics for the free and Mg^2+^ bound forms of 67RuvC

	Form 1 (free protein)	Form 2 (Mg^2+^-bound)
Data collection		
Space group	P 2_1_ 2_1_ 2_1_	P 2_1_ 2_1_ 2_1_
Cell dimensions		
a, b, c (Å)	63.1, 66.9, 73.9	66.7, 67.0, 71.1
Resolution (Å)	33.5–1.7 (1.8–1.7)	48.8–1.8 (1.9–1.8)
No. unique reflections	36621 (5282)	29210 (3894)
R_p.i.m._	0.026 (0.21)	0.043 (0.242)
Mean (I/σI)	16.3 (3.3)	8.1 (2.4)
Completeness	99.9 (99.8)	94.5 (88.3)
Multiplicity	7.2 (7.0)	3.3 (3.1)
Refinement		
Resolution (Å)	33.5–1.7	21.1–1.9
R*_work_*/R*_free_* (%)	19.7/23.7	20.2/25.4
No. atoms – Protein	2534	2538
– Solvent (Mg^2+^)	309 (0)	162 (3)
R.m.s. deviations		
Bond lengths (Å)	0.022	0.013
Bond angles (°)	1.9	1.2

Values in parentheses correspond to the highest resolution shell in the analysis of the data.

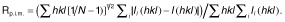

**Fig. 1:**
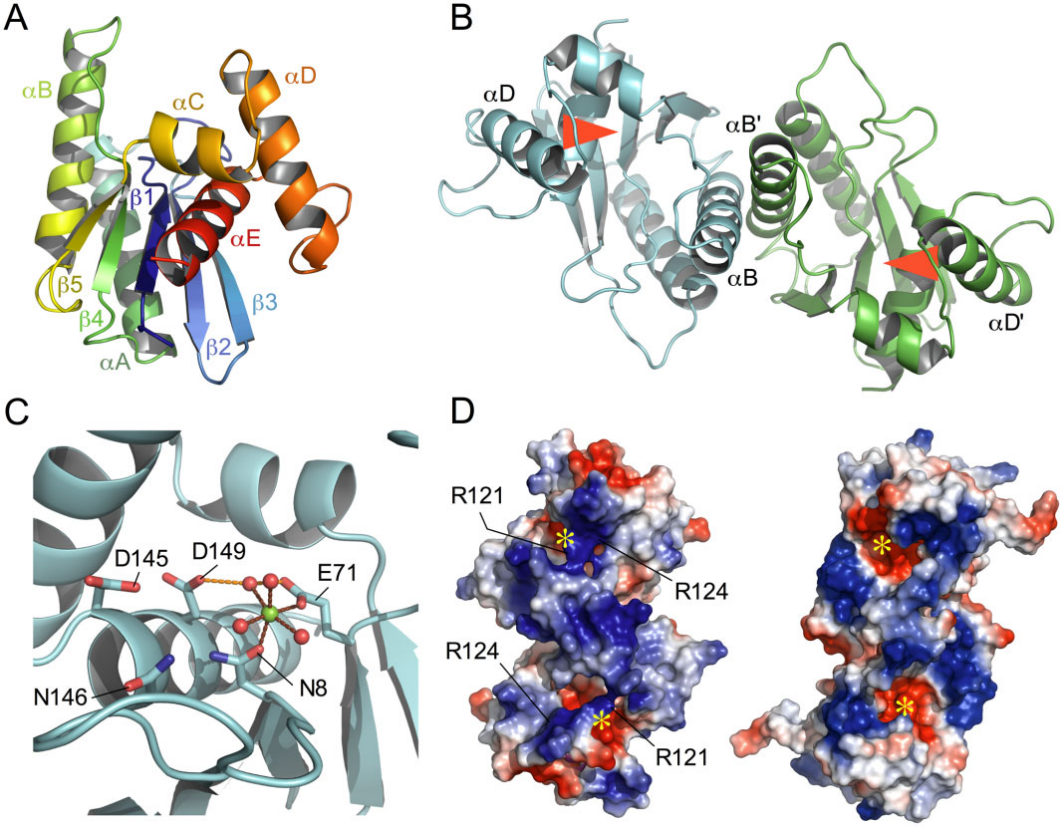
Crystal structure of 67RuvC. A. Structure in cartoon format of a monomer showing α-helices as coils and β-strands as arrows coloured in rainbow format blue-red from N- to C-terminus. Secondary structure elements are labelled. B. Structure in cartoon format as in (A) but showing a dimer with monomers coloured cyan and green. The helices forming most of the interface are labelled (αB and αB′) and the active sites are indicated by a red arrow. C. An active site depicting the relative locations of key catalytic residues (side-chains shown in stick representation) and the binding of a Mg^2+^ cation (green sphere) with additional solvent molecule ligands (red spheres). The ligand bonds to the metal and between the side-chains and the solvent are shown as red and orange dotted lines respectively. N8 is an aspartic acid in the wt RuvC structure. D. Qualitative depiction of the electrostatic surface potential of 67RuvC (left) and *Ec*RuvC (right) along the dimer twofold axes. Generated using the simple vacuum electrostatics option in PyMOL with any missing side-chains incorporated with common rotamer conformations. The charge distribution for 67RuvC is not perfectly twofold symmetric, reflecting calculations based on a non-crystallographic dimer in the asymmetric unit. The surface is coloured with red being negative, white net neutral and blue positive charge. The active sites are marked with a yellow asterisk (partially obscured in 67RuvC) and residues R121 and R124 in 67RuvC are indicated.

The 67RuvC enzyme has the classical RNase H fold common to many nucleases and integrases including its cellular *Ec*RuvC counterpart which comprises a central 5-stranded mixed β-sheet (β1–β5) flanked on one side by 2 α-helices (αA and αB) and by 3 on the other (αC, αD and αE; Fig. 1A). A non-crystallographic dimer is clearly observed in the asymmetric unit of the crystal (Fig. 1B) consistent with symmetrical resolution of Holliday junctions *in vitro* (Curtis *et al*., 2005) and the previous cellular RuvC structures (Ariyoshi *et al*., 1994; Chen *et al*., 2013). The dimer interface is formed largely by contacts along helix αB, which are predominantly hydrophobic and include the stacking of Y91 in αB with Y103 from β5 but are also supplemented by six hydrogen bonds.

Like *Ec*RuvC, 67RuvC cleaves a Holliday junction by making two near simultaneous cuts in the DNA phosphodiester backbone, one catalysed by each active site in the two monomers of the dimer. The key catalytic residues (D8, E71, D145 and D149) postulated to hold an activating Mg^2+^ ion in place were identified by sequence alignment with the well-defined active site in cellular RuvC (Curtis *et al*., 2005). The structures presented here, where D8 has been mutated to an asparagine, confirm that these residues are indeed clustered together in a pocket recessed slightly from the surface of the protein (Fig. 1C), which is partially obscured by a loop formed by residues 12–16. A structure of 67RuvC has also been determined with one of the two expected Mg^2+^ atoms bound in each active site pocket (Fig. 1C). The octahedral co-ordination geometries and distances (2.2 Å to 2.0 Å) between the modelled Mg^2+^ cations and the carboxyamide and carboxyl groups of N8 and E71 in the two monomers support the identity of the bound cations. The D149 residue in each monomer also contacts a water molecule, which could serve as another co-ordinating ligand; the remaining co-ordination points are made by waters. The fourth catalytically implicated residue, D145, is comparatively distant from the Mg^2+^ ion with about 8 Å between the cation ion and Cγ of the side-chain. Overall there is no major difference between the structures with and without Mg^2+^.

A simple electrostatic surface calculation providing a qualitative description of the surface potential of the 67RuvC dimer shows that there is a marked difference between the face containing the active site pockets and that on the other side of the protein (Fig. 1D). Examination of the charge distribution on the surface of 67RuvC shows that the negatively charged active site pockets are surrounded by a notably positive electrostatic potential. This positive charge is evenly distributed over the surface, consistent with binding a negatively charged DNA backbone that could then gain access to the recessed pockets containing the catalytic residues. Interestingly, our simple analysis suggests that 67RuvC shows a more pronounced overall positive charge than that observed on the junction-binding surface of *Ec*RuvC (Fig. 1D).

### Comparison of the bIL67 RuvC structure with bacterial RuvC and yeast mitochondrial Holliday junction resolvases

**Fig. 2:**
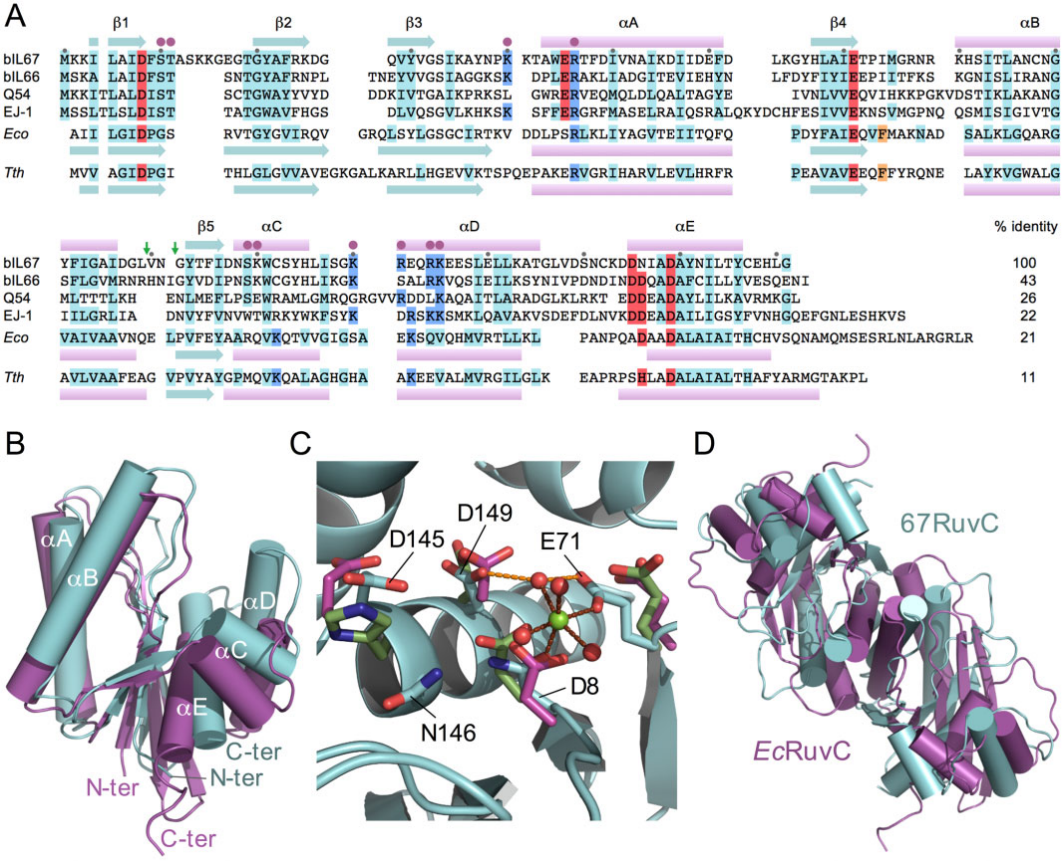
Comparison of phage and cellular RuvC resolving enzymes. A. Alignment of selected phage RuvC proteins. Structural elements of phage bIL67, *Escherichia coli* (*Eco*) and *Thermus thermophilus* (*Tth*) proteins are indicated above and below the aligned sequences (pink bars, α-helix; cyan arrows, β-sheet). Conserved residues are highlighted in red (acidic; active site), blue (basic), cyan (others) and orange (F69 in *Ec*RuvC and F73 in *Tth*RuvC). Every ten residues are labelled as filled grey circles above the bIL67 RuvC sequence. Filled magenta circles indicate the location of bIL67 RuvC S10A, T11A, K40A, R46A, S109A, K110A, K120A, R121A, R124A, K125A and R124A+K125A substitution mutants. Sequence conservation in *E. coli* and *T. thermophilus* RuvC is taken from the Pfam database entry, PF02075, highlighting the most highly conserved residues among bacterial RuvC family proteins. Conserved residues in phage RuvC are based on 24 orthologues using a similar approach. The percentage identity refers to homology of each protein with bIL67 RuvC. Green arrows indicate the positions of insertions to generate N- and C-terminal 67-*Ec*RuvC and *Ec*-67RuvC hybrids (see Fig. S6). Accession numbers of selected lactococcal phage RuvC proteins from bIL67 (NP_042322), bIL66 (AAA99046), Q54 (YP_762587) and *Streptococcus pyogenes* inducible phage EJ-1 (NP_945263). B. Structural superimposition based upon their central β-sheets of monomers of 67RuvC (cyan) and *Ec*RuvC (dark magenta) shown in cartoon representation as in Fig. 1 but with α-helices shown as cylinders. Helices and termini are labelled. C. Superimpostion of the catalytically critical residues at the active sites of 67RuvC (cyan), *Ec*RuvC (dark magenta) and *Tth*RuvC (olive green). Side-chains are shown in stick representation with the rest of the structure in cartoon form. The 67RuvC residue labelled D8 is an asparagine in the crystal structure shown. The Mg^2+^ cation (green sphere) and solvent ligands (red spheres) from the 67RuvC structure are shown for reference. D. Alignment of dimers of 67RuvC and *Ec*RuvC based upon superimposition of the αB dimer interface helices. Structures are represented and coloured as in (B).

A comparison of the phage 67RuvC structure with that of cellular *E. coli* and *T. thermophilus* RuvC (*Tth*RuvC) proteins (Protein Data Bank entries 1HJR and 4EP4 respectively) reveals significant differences in the relative arrangement of the secondary structure elements in 67RuvC. The structures of the bacterial enzymes are very similar as noted elsewhere (Chen *et al*., 2013) and thus we will largely restrict the comparison to that of 67RuvC with *Ec*RuvC. Superimposition of a monomer from each of the structures based upon alignment of the central 5-stranded β-sheet can be seen in Fig. 2B. It shows that the αB helices superimpose well, however, helices αC, αD and αE in 67RuvC do not. These three helices are expected to create the DNA binding sites in the resolvase enzymes and are markedly different in their relative orientations in the structures.

Despite the poor relative alignment of the core β-sheet and helix αD within a monomer, when considering a more localized superimposition using only the catalytically critical residues from a single monomer in each enzyme (*Ec*RuvC residues D7, E66, D138, D141, *Tth*RuvC D7, E70, H143, D146 and 67RuvC residues N8(D8), E71, D145, D149), a similar arrangement of these residues is observed (Fig. 2C). The RMSD for the superimposition of the alpha carbon atoms is 1.4 Å. A more detailed inspection shows that three of the four side-chains, located on strands β1 and β4 and helix αD, adopt very similar conformations and closely superimpose. However, *Ec*RuvC D138, *Tth*RuvC H143 and 67RuvC D145 do not align well and clearly the effect of changes in relative positions of the core β-sheet and helix αD have not been compensated for in this case.

Analyses of the overall structures of the resolvases via superimpositions of dimers of 67RuvC and *Ec*RuvC based upon the αB interface helices (Fig. 2D) reveal a considerable relative rotation of the monomers about the dimer interface. This affects in particular the relative locations within a dimer of the αD helices and extends to the alignment of the core β-sheets.

There is another RuvC-like structure in the Protein Data Bank, the *Schizosaccharomyces pombe* mitochondrial resolvase Ydc2 (entry 1KCF; Ceschini *et al*., 2001). Ydc2 has DNA structure and sequence specificities that resemble those found with *E. coli* RuvC (Oram *et al*., 1998). Superimpositions of 67RuvC and the yeast enzyme (Fig. S2) reveal more extensive differences than those noted between 67RuvC and the bacterial RuvC proteins and these are explored further in the legend to Fig. S2.

### Model for Holliday and fork binding

**Fig. 3:**
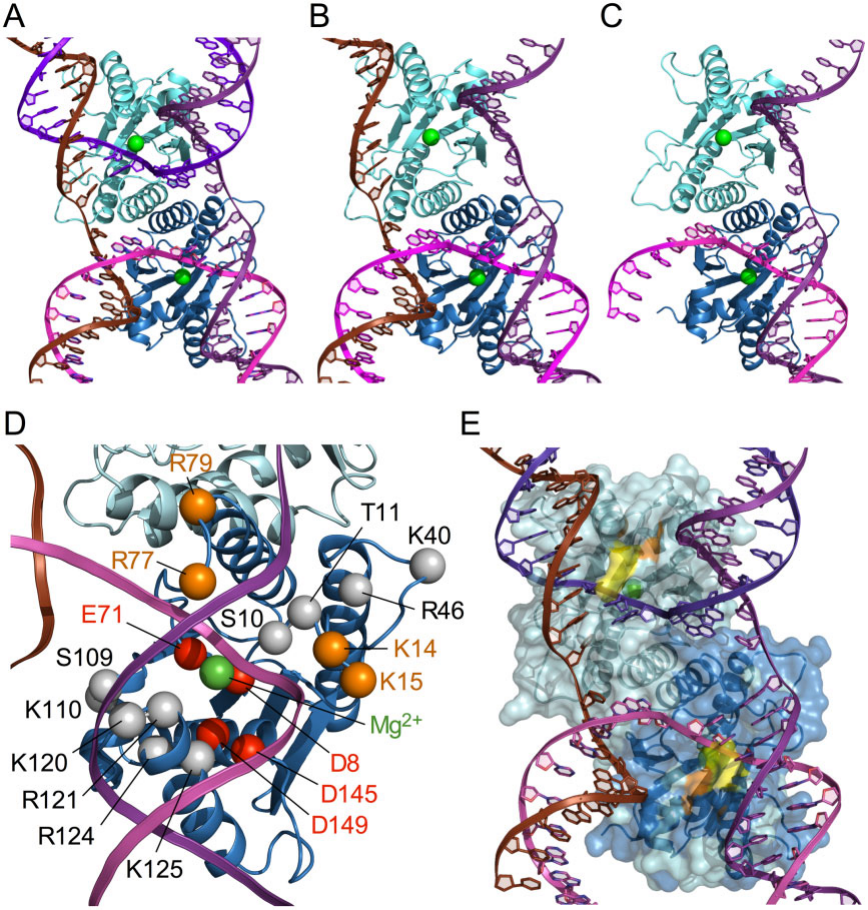
Models for 67RuvC bound to Holliday, flap and fork junctions. A. A dimer of 67RuvC and a Holliday junction model based on that of the T4 endo VII-DNA complex (Biertumpfel *et al*., 2007). 67RuvC and DNA are shown in cartoon versions with the protein remote from the viewer with monomers coloured cyan and blue, bound Mg^2+^ cations as green spheres and the four strands of the DNA coloured brown, purple, magenta and violet with the trace of the phosphate backbone represented by a ribbon. B. A model of a complex with a 3-stranded flap junction. C. A model of a complex with a 2-stranded fork junction. D. The locations of key residues within a monomer of 67RuvC. Alpha carbon positions are highlighted with spheres (grey for those residues mutated in this study; red for the catalytically important, cation binding residues and orange for other residues referred to in the text), labelled and shown relative to the modelled DNA as in Fig. 3A. The location of the bound Mg^2+^ cation is also shown with a green sphere. E. A model of a complex with a 4-stranded Holliday junction highlighting the location of the R121A (yellow) and R124A (orange) mutants studied *in vitro*.

Thus far it has not been possible to produce crystals of a 67RuvC complex with a DNA substrate, as with all RuvC family proteins studied to date, and thus simple models have been constructed for the possible binding of the enzyme to branched DNA (Fig. 3). This approach facilitated the selection of residues for testing, which might have a functional role either in DNA binding or substrate selectivity. These models have been chosen from the many potential arrangements of the arms in branched DNA junctions derived from both known structures and a large range of theoretical models. The selection was based upon docking the components manually so as to minimize the number of possible clashes between protein and DNA while balancing this against knowledge of the active site locations and the distances required for attack of an activated nucleophile on the DNA phosphate backbone. In constructing the models, conformational changes in the protein and/or local deformation of the phosphate backbone were recognized as possible but could not be reliably incorporated. The obvious features of the DNA-binding surface of 67RuvC are the P73-N78 loops, the αB helices at the dimer interface and the grooves formed between them and the αD helices in which the active site pockets are located. Thus models for the DNA component, which had a more ‘open’ central region between the arms of the junction, could be bound more readily with fewer clashes. This fits with experimental evidence showing that assembly of *Ec*RuvC onto Holliday junction DNA yields an unfolded structure with twofold symmetry (Bennett and West, 1995). The best model for a 67RuvC-DNA Holliday junction complex was based upon the experimentally determined structure of a 4-way junction obtained from the phage T4 endo VII-DNA complex (Biertumpfel *et al*., 2007). Examination of the model shows that the central region of 67RuvC protrudes into the hole in the molecular surface at the middle of the DNA junction and the N-termini of the αD helices lie in the major groove of opposing DNA arms (Fig. 3A). In this model the exchanging strand, as defined for a stacked-X junction (Churchill *et al*., 1988), would be cleaved. This is also observed for phage T4 endo VII but is in contrast to that predicted for *Ec*RuvC (Bennett and West, 1995), where the continuous strand is cleaved in an assay with a constrained junction. The directly equivalent assay has not been performed with 67RuvC and thus an alternative model in which the DNA junction might be cut by 67RuvC on its continuous strands was generated (not shown). In this model the fit of protein and DNA is not as good but again we recognize that unforeseen conformations might occur in either component. *In vivo*, *Ec*RuvC is postulated to function in a resolvasome with the RuvAB translocase (Sharples *et al*., 1999) where the DNA junction would be held in a fourfold symmetric conformation and there is no distinction between continuous and exchanging strands.

The active site pockets are positioned relative to the DNA phosphate backbone so as to enable attack by a water molecule activated by the Mg^2+^ cations bound by the acidic aspartate and glutamate side-chains. Indeed the co-ordination shell of one or both of the bound cations might be completed by the DNA phosphate backbone, which could displace cation-bound water molecules and position itself for attack by an activated water. The most likely scissile bond to be cleaved in this model would be two pairs removed from the cross-over point, consistent with previous data on Holliday junction cleavage by 67RuvC (Curtis *et al*., 2005).

Residues that come close to the DNA in the junction include R77 and R79 (Fig. 3D), which protrude into the junction centre. Their positive charges may indicate an interaction with phosphate groups in the open centre of the Holliday junction. The electron density for the side-chains of these residues is weak in the free enzyme structure but it is quite possible that these residues would become ordered when the protein is in complex with the DNA substrate.

Residues at the N-terminal end of αD and the preceding turn are also in close proximity to the DNA in the model and this includes K120, R124 and K125, which are conserved in phage RuvC evolution, and R121 (Figs 2A, 3D and E). Residue K125 is equivalent to the very highly conserved K118 found in *Ec*RuvC and other members of the RNase H superfamily (Yoshikawa *et al*., 2000). Major differences exist between *Ec*RuvC and 67RuvC in this region with regards both to the sequence and the structure of the turn, which may have an impact on the binding selectivity or specificity of 67RuvC. Helices αC, αD and the intervening turn are highly conserved in bacterial RuvC proteins and are postulated to fulfil a role in Holliday junction recognition and sequence specificity of resolution (Hagan *et al*., 1998; Yoshikawa *et al*., 2000). As suggested previously from sequence alignments (Curtis *et al*., 2005), there is no residue positioned in the 67RuvC structure equivalent to *Ec*RuvC F69 or *Tth*RuvC F73, which is believed to carry out a role in stacking with the DNA nucleobases as part of the source of sequence specific cleavage (Yoshikawa *et al*., 2001; Chen *et al*., 2013). Similarly there are no large aromatic residues in 67RuvC equivalent to F74 and Y75 also previously highlighted in *Tth*RuvC (Chen *et al*., 2013).

67RuvC is known to process DNA branch points other than Holliday junctions, like similar phage resolvases but unlike *Ec*RuvC. Thus, using the 4-stranded Holliday junction with its four duplex DNA arms as a guide, models were constructed of 2- and 3-stranded DNA substrates with either 1 (fork; Fig. 3C) or 2 (flap; Fig. 3B) duplex arms plus flexible single-strand tails, as tested *in vitro* (see below; Curtis *et al*., 2005). Further models were then built of possible complexes with 67RuvC. Junctions constructed with only one or two duplex arms are capable at any given moment of fully contacting only a single DNA binding cleft formed by one monomer in an enzyme dimer. Thus their interaction superficially resembles the binding to duplex DNA where the DNA can only be bound at its ends because of clashes with the central region of the enzyme dimer and consequently far fewer contacts are made relative to a Holliday junction. However, the presence of the additional duplex arm in the 3-stranded flap and single-stranded tails in both the fork and flap junctions do allow for extra interactions between the DNA and residues in the protein (Fig. 3B). Residues in the loop preceding αB, its N-terminus and the N-terminal residues of αD are well positioned to make possible contacts with the proximal end of the second duplex arm in the flap substrate and with the single stranded tails in this and the fork substrate (Fig. 3B and C). Indeed these tails might be capable of extending around into the active site region of a second monomer. This may account for 67RuvC incisions on fork substrates located 4–8 nucleotides from the branch point (Curtis *et al*., 2005), although it should be noted that the strand polarity would be opposite to that normally found when bound to a 4-stranded X junction unless further looping back of the DNA strand is involved. The additional positive charges in the vicinity of αD in the 67RuvC structure may stabilize DNA interactions and help explain its ability to bind a fork substrate almost as well as a Holliday junction, and much better than that of duplex DNA, which is distinctly different to the situation with *Ec*RuvC where fork substrates are bound as poorly as duplex DNA when compared with a Holliday junction (Curtis *et al*., 2005).

### Site-directed mutagenesis of bIL67 RuvC

To help identify features in 67RuvC responsible for Holliday junction and replication fork recognition, eleven site-directed mutants in conserved, polar residues were generated. The mutants, S10A, T11A, K40A, R46A, S109A, K110A, K120A, R121A, R124A, K125A and a double mutant R124A+K125A flank the active site and project from the walls of the DNA junction binding cleft (Fig. 3D and E). S10 and T11 lie at the bottom of this groove in each subunit, close to D8 and other catalytic residues that comprise the active site and are invariant in 24 phage RuvC proteins (data not shown), while R46 and K40 lie more remote from the proposed DNA binding interface (Fig. 3D). The remaining residues are located in the αC–αD region, which differs significantly from bacterial RuvC proteins (this work; Curtis *et al*., 2005). Two adjacent basic residues point into the DNA binding cleft (R124 and K125). K120 is also conserved and projects out into the solvent. R121 is less well conserved although adjacent positively charged residues in other phage RuvC proteins could compensate for its absence (Fig. 2A).

### Nicking of replication forks by 67RuvC mutants *in vivo*

**Fig. 4:**
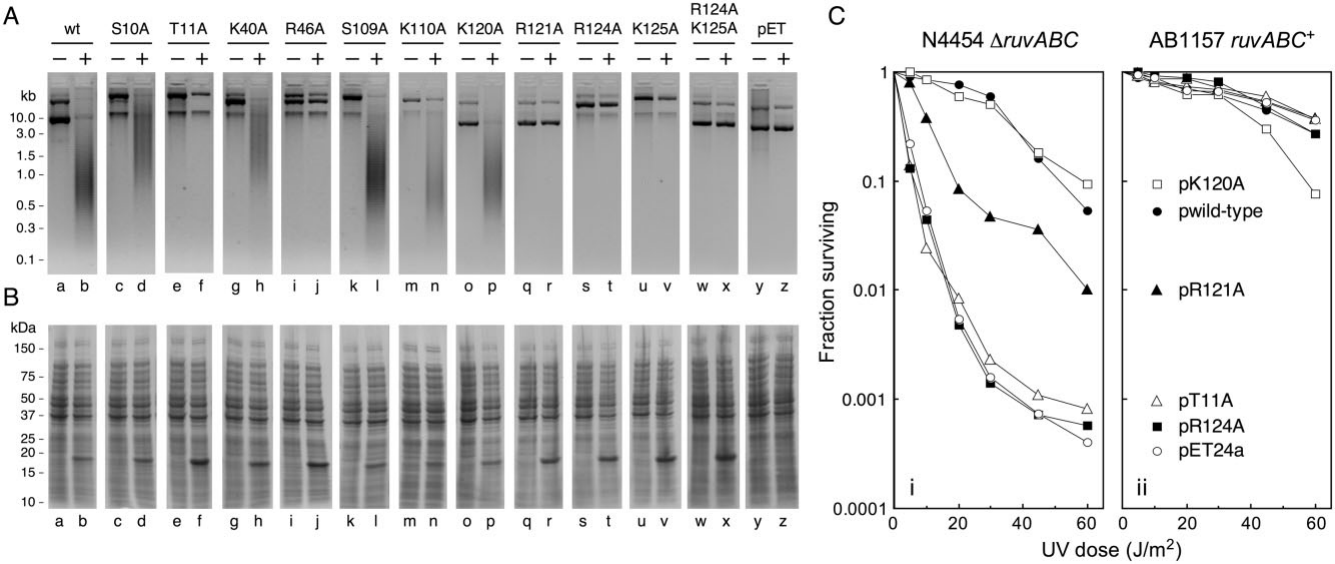
*In vivo* analysis of 67RuvC mutant protein expression, fork cleavage and Holliday junction resolution. A. Formation of chromosome and plasmid DNA breaks by 67RuvC. BL21-AI strains carrying wild-type (wt) or mutant bIL67 *ruvC* genes in the T7 expression vector pET24a (pET) were grown to an A_650nm_ of 0.6. Expression of 67RuvC was induced in half of the culture by addition of arabinose and IPTG (+), while the other half served as an uninduced control (−). Cells were harvested after a further hour of growth and DNA isolated before loading on a 1% agarose gel containing ethidium bromide. B. Analysis of levels of 67RuvC expression. Uninduced and induced cells were prepared as in (A). Total cellular protein was analysed by separation on 12.5% SDS-PAGE and visualized by staining with Coomassie blue. C. Holliday junction resolution by 67RuvC mutants *in vivo*. Strain and relevant genotypes are indicated above each panel and the UV light survival of selected mutants shown alongside wt 67RuvC and pET24a(+) control plasmids.

Both bIL66 and bIL67 RuvC proteins induce the formation of chromosomal breaks in *E. coli* due to nicking of the branched structures formed during DNA replication (Bidnenko *et al*., 1998; Curtis *et al*., 2005). To monitor the effect of the various bIL67 RuvC mutants on chromosomal fragmentation *in vivo*, we introduced plasmids carrying the wt or mutant 67*ruvC* gene into BL21-AI strains. This background permits high-level gene expression of the target protein from a T7 promoter upon addition of arabinose and IPTG. DNA breakage was assayed in induced and uninduced cells by isolating plasmids in conjunction with any fragmented chromosomal DNA; commercial plasmid purification protocols retain linear and circular DNA molecules up to 150 kb in size. As noted previously (Curtis *et al*., 2005), expression of 67RuvC generated significant smearing of the recovered DNA in the induced but not uninduced cultures (Fig. 4A, lane a and b). The degraded material corresponds to fragmented and linearized chromosomal and plasmid DNA. In fact plasmid material is largely eliminated by 67RuvC expression (Fig. 4A, lane b). Similar results were obtained with constructs expressing S109A, K110A and K120A mutant proteins (Fig. 4A, lanes k–p), suggesting that these mutations do not significantly impair the ability of 67RuvC to cut replication forks. The S10A and K40A mutants also produced DNA fragmentation (Fig. 4A, lanes d and h), although the smear consisted of bands with a greater molecular mass perhaps indicating a slight reduction in the branch nicking activity of these mutants. The remainder of the plasmids carrying 67RuvC mutations failed to generate any DNA breakages when expression was induced, matching the results obtained with the pET24a vector control (Fig. 4A). These results are consistent with a defect in nicking activity as a consequence of the T11, R46, R121, R124 and K125 alanine substitutions.

In parallel with the plasmid and chromosomal fragmentation analysis, 67RuvC expression was evaluated by separating total cellular proteins by SDS-PAGE (Fig. 4B). The results follow a similar pattern to that seen with the fork cleavage analysis. Plasmids expressing a catalytically active protein (Fig. 4B, lanes b, d, h, l, n and p) showed reduced expression of the 18 kDa product relative to those that failed to produce DNA smearing (Fig. 4B, lanes f, j, r, t, v and x). This constrained expression is most likely a consequence of ongoing plasmid degradation following overproduction of the 67RuvC fork nicking activity. Those mutants with a marked defect in forming DNA breaks (T11A, R46A, R121A, R124A, K125A and R124A+K125A) show enhanced levels of expression because plasmids remain undamaged. The latter results match those seen previously with the 67RuvC D8N active site mutant, which is defective in both fork and Holliday junction cleavage (Curtis *et al*., 2005).

### Holliday junction resolution by 67RuvC mutants *in vivo*

Plasmids carrying 67RuvC are capable of suppressing the UV sensitive repair phenotype of an *E. coli ruvABC* mutant (Fig. 4C; Curtis *et al*., 2005), confirming that the phage enzyme functions as a Holliday junction resolvase *in vivo*. The deleterious plasmid and chromosomal fragmentation observed in BL21 strains does not occur because the T7 promoter in pET24a(+) is inoperative in this genetic background; sufficient but limited 67RuvC expression must arise from weak promoters located elsewhere in the vector. The Holliday junction resolution activity of 67RuvC mutants was examined by assaying the UV light sensitivity of a *ruvABC* resolution-defective strain carrying each construct. A *ruvABC*^+^ strain of the same lineage was used as a control to assess any deleterious effect of protein expression in the presence of an intact RuvABC system. The 67RuvC wt restored UV resistance to the *ruvABC* mutant, as did mutants S10A, K40A, S109A, K110A and K120A (Fig. 4C and Table 2). These five mutant proteins therefore behave much like wt 67RuvC in both replication fork and Holliday junction assays *in vivo*. In contrast, the T11A, R46A, R124A, K125A, R124A+K125A mutants resembled the vector control in their inability to improve the UV resistance of the *ruvABC* mutant strain (Table 2), suggesting they are substantially or entirely deficient in cleaving branched junctions. The remaining mutant, R121A, restored partial UV resistance to a *ruvABC* strain, indicating that it retains some Holliday junction resolution activity *in vivo* (Fig. 4C; Table 2). This is particularly noteworthy since no fork cleavage activity was detected in the plasmid assays with this mutant (Fig. 4A, lane r). Hence R121A could potentially represent a separation of function mutant that shows impaired recognition of replication fork structures, while preserving Holliday junction resolvase activity; analogous to converting the phage enzyme to one that resembles cellular RuvC family endonucleases.

**Table 2:** Effect of plasmids carrying mutations in 67RuvC on the survival of UV-irradiated *ruvABC* and *ruv^+^* *E. coli* strains

Strain	Fraction surviving (30 J m^−2^)	

	N4454	AB1157
Genotype	Δ*ruvABC*::*cat*	*ruvABC*^+^
wt	0.60 ± 0.005	0.62 ± 0.015
S10A	0.61 ± 0.010	0.49 ± 0.030
T11A	0.0023 ± 0.0012	0.69 ± 0.015
K40A	0.67 ± 0.010	0.79 ± 0.030
R46A	0.0026 ± 0.00015	0.80 ± 0.015
S109A	0.46 ± 0.0050	0.60 ± 0.075
K110A	0.52 ± 0.0050	0.78 ± 0.050
K120A	0.50 ± 0.0010	0.62 ± 0.115
R121A	0.068 ± 0.022	0.71 ± 0.012
R124A	0.0014 ± 0.00025	0.81 ± 0.050
K125A	0.0014 ± 0.0011	0.41 ± 0.060
R124A+K125A	0.0014 ± 0.00005	0.80 ± 0.025
pET24a	0.0016 ± 0.00020	0.66 ± 0.040

bIL67 *ruvC* and mutant derivatives cloned in pET24a(+) and relevant strain genotypes are indicated. Results are the mean and standard deviation of at least two independent experiments.

Neither wt nor mutant 67RuvC clones significantly increased the UV light sensitivity of *ruvABC*^+^ strains (Fig. 4C; Table 2). The results with the wt, DNA repair-proficient strain are consistent with few, if any, chromosome breaks, minimal levels of 67RuvC expression and no major deleterious effects upon cell survival.

### DNA branch selectivity of 67RuvC R121A and R124A mutants

**Fig. 5:**
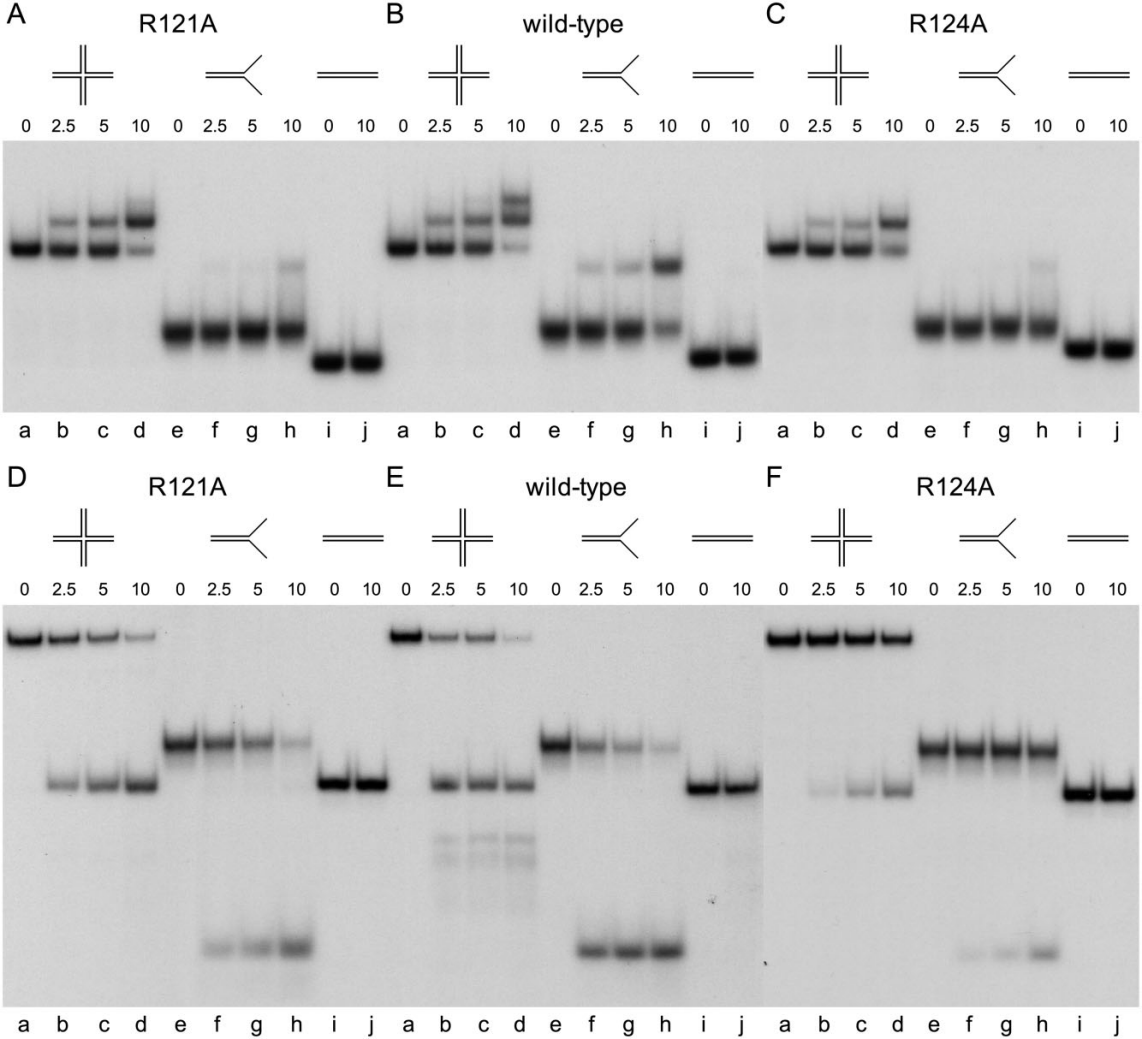
Branched DNA binding and cleavage by 67RuvC R121A and R124A mutant proteins. A–C. Gel retardation assay showing binding of R121A, R124A and wt 67RuvC to junction (J11, lanes a–d), fork (F11, lanes e–h) and duplex (D11, lanes i–j) DNA substrates. Binding reactions contained 5 mM EDTA, 0.3 nM ^32^P-labelled DNA and protein at 2.5, 5 and 10 nM. Samples were incubated on ice for 15 min before separation on 4% PAGE. Lanes a, e and i served as no protein controls. D–F. DNA branch cleavage assay showing the resolution products of R121A, R124A and wt 67RuvC on junction (J11, lanes a–d), fork (F11, lanes e–h) and duplex (D11, lanes i–j) DNA substrates. Reactions contained 10 mM MgCl_2_, 0.3 nM ^32^P-labelled DNA and protein at 2.5, 5 and 10 nM. Lanes a, e and i served as no protein controls. Reactions were incubated for 15 min at 37°C before processing and separation on 10% PAGE.

**Fig. 6:**
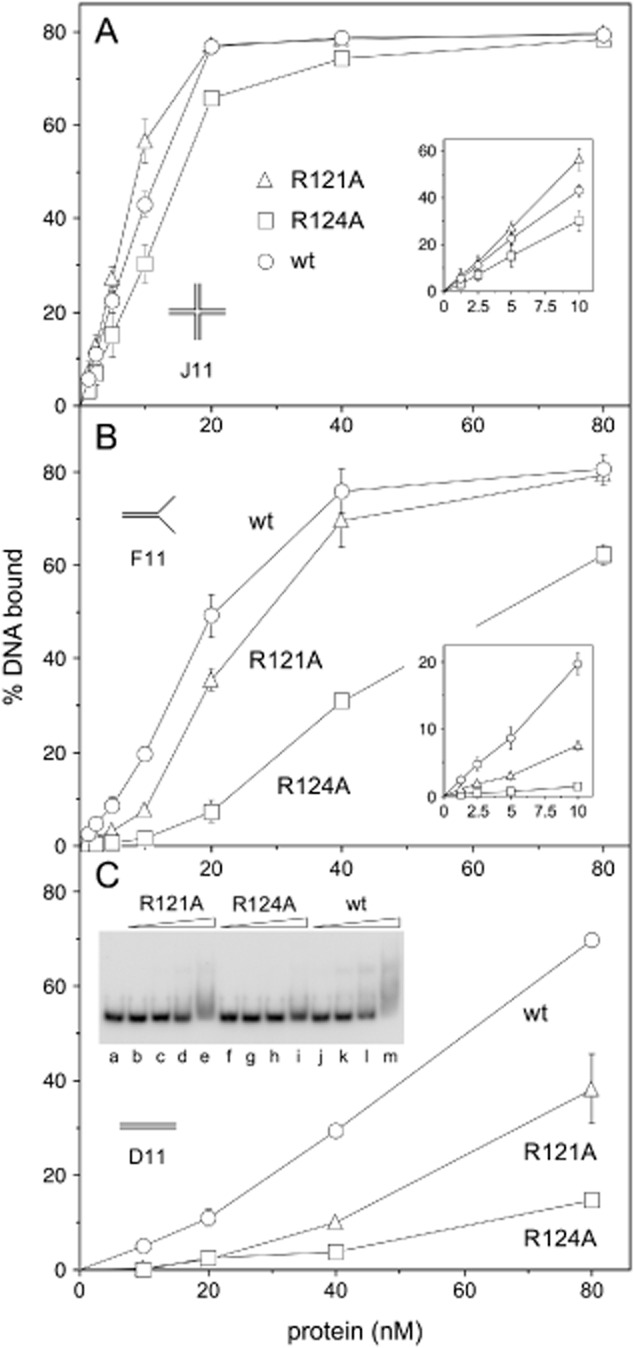
Comparison of 67RuvC R121A and R124A mutant protein binding to Holliday junction, fork and duplex DNA substrates. Binding reactions contained 5 mM EDTA, 0.3 nM ^32^P-labelled J11 (A), F11 (B) and D11 (C) and protein at 1.25, 2.5, 5, 10, 20, 40 and 80 nM in (A) and (B); only the latter four concentrations were used in (C). Samples were incubated on ice for 15 min before separation on 4% PAGE and gels analysed by phosphorimaging. Data are the mean and standard deviation of two independent experiments.

To determine whether the *in vivo* properties of 67RuvC R121A genuinely corresponded to an alteration in structure-selectivity, the mutant protein was purified to homogeneity and its junction binding and cleavage activities evaluated on ^32^P-labelled DNA substrates *in vitro* (Fig. 5). Another mutant, R124A (located close to R121 in αD; Fig. 3D and E) and apparently defective in both replication fork and Holliday junction cleavage (Fig. 4), was also purified and assayed in parallel. In gel shift assays at lower concentrations of protein, the R121A and R124A mutants formed a single complex with a model 50 bp Holliday junction (J11) containing an 11 bp core of homology (Fig. 5A and C, lanes b–d). R124A showed a modest reduction in junction binding with a *K_D_* of 1.21 × 10^−8^ M relative to the *K_D_* for the wt protein of 9.53 × 10^−9^ M (Fig. 5B and C, lanes b–d; Fig. 6A), whereas R121A protein displayed a slightly higher affinity, 7.33 × 10^−9^ M for the X junction (Fig. 6A). Additional complexes were observed at higher protein concentrations with both wt and mutant proteins (Fig. 5B and data not shown). However, formation of only a single protein–junction complex was favoured with the mutant proteins, suggesting that loss of a single positive charge in this region destabilizes interactions with linear DNA. The additional distinct complexes formed on Holliday junction DNA (Fig. 5B, lane d) probably reflects assembly on each junction arm (Curtis *et al*., 2005), although on a 50 bp linear duplex, wt 67RuvC bound poorly and formed less stable complexes as judged by extensive band smearing (Fig. 6C, lane m). Some cooperativity in junction binding is therefore indicated, in keeping with the sigmoidal binding curves observed in Fig. 6. The mutant proteins, especially R124A, bound the linear DNA even more weakly than the wt (Fig. 6C). Thus the modifications introduced into 67RuvC bestow a substantially improved DNA branch specificity relative to the wt enzyme. The mutant junction binding specificity may actually be considerably higher as the enhanced capacity of the wt to assemble on duplex DNA will artificially raise its affinity for branched substrates.

A 50 bp fork junction (F11), made by omitting two of the oligonucleotides from J11 and resembling a replication fork structure, was also used in DNA binding assays. R124A showed reduced affinity for this substrate (*K_D_* of 4.57 × 10^−8^ M), whereas the wt and R121A bound comparatively well with apparent dissociation constants of 1.75 × 10^−8^ M and 2.20 × 10^−8^ M respectively (Fig. 5A–C, lanes f–h and Fig. 6B). R124A, which exhibited only a slight reduction in binding to J11, displayed the poorest binding of the two mutants, especially at lower protein concentrations (Fig. 6B, inset). However, R121A did also show reduced binding to F11 at lower protein concentrations, whereas it bound better at these concentrations to J11 DNA (Fig. 6A and B insets).

To further probe the DNA branch selectivity of R121A and R124A, two additional fork structures were tested, one resembling a Y junction with fully double-stranded arms (F12-Y) and another that more closely mimics a genuine replication fork structure, with leading strand present and lagging strand omitted (F12-RF; Fig. S3). 67RuvC wt bound well to these two substrates, showing a slight preference for F12-Y (Fig. S3A and B, lanes n–s). Binding to F12, an equivalent of the F11 fork, was comparable to that seen with F12-RF (Fig. S3B and C, lanes n–s). The provision of additional duplexes in F12-RF and F12-Y, rather than the single duplex present in F12, facilitated the formation of additional protein-DNA complexes (Fig. S3A–C, lanes n–s). Thus the multiple complexes observed with 67RuvC on Holliday junction DNA (Curtis *et al*., 2005) most likely arise by sequential addition of dimers on accessible duplexes, with ssDNA being insufficient to promote stable assembly. Both R121A and R124A bound fairly well to F12-Y, with the former showing a similar binding profile to that displayed by the wt protein (Fig. S3A and D). The pattern of binding is not too dissimilar to that observed with J11 (Fig. 6A) and indicates that this fork mimics more closely a Holliday junction, with the three duplexes helping to stabilize binding even with the two mutant derivatives. The considerably more selective *E. coli* RuvC enzyme is known to form unstable complexes with related Y junction substrates (Takahagi *et al*., 1994), consistent with such structures resembling a 4-way junction closely enough to support limited binding.

In contrast to F12-Y, R121A and R124A bound relatively poorly to F12-RF (Fig. S3B), displaying similarly weak binding to that seen with the F12 fork (Fig. S3C). R124A bound especially poorly to these two forks (Fig. S3B and C, lanes h–m), despite binding well to F12-Y (Fig. S3A, lanes h–m). Both of the mutants form unstable complexes as judged by band smearing, whereas the wt forms discrete complexes (Fig. S3B). Given that F12-RF matches most closely a replication fork, it is evident that the two 67RuvC mutants significantly impair stable interactions with fork structures that incorporate ssDNA in one or both strands, while exhibiting only minor defects in binding to fully double-stranded Y and X junctions. Hence, these positively charged residues located in the upper, outer walls of the junction binding cleft of 67RuvC play an important role in DNA branch recognition, ensuring that structures that resemble replication forks can be accommodated and cleaved.

To probe any changes in branch resolution activity, the assays were repeated under conditions suitable for 67RuvC endonuclease activity by incorporating magnesium ions and incubating reaction mixtures at 37°C. Mutant and wt proteins cleaved the Holliday junction substrate to generate a product migrating at the same position as the linear duplex (Fig. 5D–F, lanes b–d). This band is consistent with genuine Holliday junction resolution by symmetrically related paired incisions at the branch point to yield nicked duplex DNA (Curtis *et al*., 2005). R124A showed a significant reduction in X junction cleavage activity relative to the wt, whereas R121A displayed only a modest reduction (Fig. 5D–F; Fig. S4). Significantly, the mutant proteins generated fewer of the faster-migrating DNA bands evident with the wt (Fig. 5E, lanes b–d); these resolution products arise from asymmetry in strand scission to release portions of a single junction arm. Thus both mutants appear to cleave junctions with enhanced resolution symmetry. The minor defect in R121A Holliday junction resolution fits with its ability to partially resolve Holliday junctions *in vivo* (Fig. 4C).

Despite the reduced binding of R121A to F11 fork DNA (Figs 5A and 6B, inset), it only showed a slight decrease in cleavage activity compared with the wt at low protein concentrations (Fig. 5D and E, lanes f–h; Fig. S4). This reduced level of activity must be sufficient to prevent detection of replication fork cleavage *in vivo* (Fig. 4A, lane r), although it highlights the limitations of relying solely on such assays to distinguish diminished and abrogated activities. However, both *in vivo* and *in vitro* results are consistent with the R121A mutation resulting in reduced fork resolution compared with its activity on Holliday junctions. R124A exhibited a significant reduction in both Holliday junction and fork DNA cleavage relative to the wt (Fig. 5E and F). The weak nuclease activity on both branched substrates (Fig. 5F) is in keeping with the inability of R124A to function *in vivo* in either chromosomal breakage or Holliday junction resolution experiments (Fig. 4A, lane t and Fig. 4C). Similar results were obtained using forks F12-Y and F12-RF (Fig. S5), with R124A again exhibiting a much more severe defect in cleavage. Interestingly, while both mutants bound much like the wt to F12-Y (Fig. S3A and D), they cleaved this substrate less well than F12-RF (Fig. S5B). This suggests that the R121A and R124A mutants have some defect in correctly positioning the duplex arms in F12-Y for efficient resolution.

The *in vitro* cleavage data with R121A and R124A show less of a distinction in their preference for Holliday junction over fork DNA (Fig. S4) than that noted in the binding studies. Part of the reason for this discrepancy is that a single nick in fork DNA yields a distinct resolution product of smaller size, whereas at least two incisions in a Holliday junction are required to yield a product distinguishable from the intact substrate. Cleavage activity is also affected by the nucleotide sequence preference of the enzyme on each substrate. These assays therefore tend to exaggerate the activity of both wt and mutant proteins on a fork substrate and hence are less reliable indicators of DNA branch selectivity. Furthermore, the lack of asymmetrical products of Holliday junction resolution with the R121A and R124A mutants but not the wt (Fig. 5D–F) might result in a reduced cleavage activity on this substrate relative to fork DNA.

Overall, the binding and cleavage experiments suggest that arginines 121 and 124 make an important contribution to DNA branch discrimination and endonuclease activity. This is particularly evident with the R124A mutant, which binds poorly to forks containing at least one single stranded component and duplex DNA but shows only a modest reduction in binding to the Holliday and Y-shaped fork junctions. The two arginines clearly fulfil different functions with mutation of R124 conferring a much more severe defect in branched DNA cleavage. Most importantly, however, both R121A and R124A behave similarly in producing a single resolution product on a Holliday junction, consistent with a marked enhancement in resolution specificity over the wt enzyme.

### DNA sequence specificity of 67RuvC R121A and R124A mutants

**Fig. 7:**
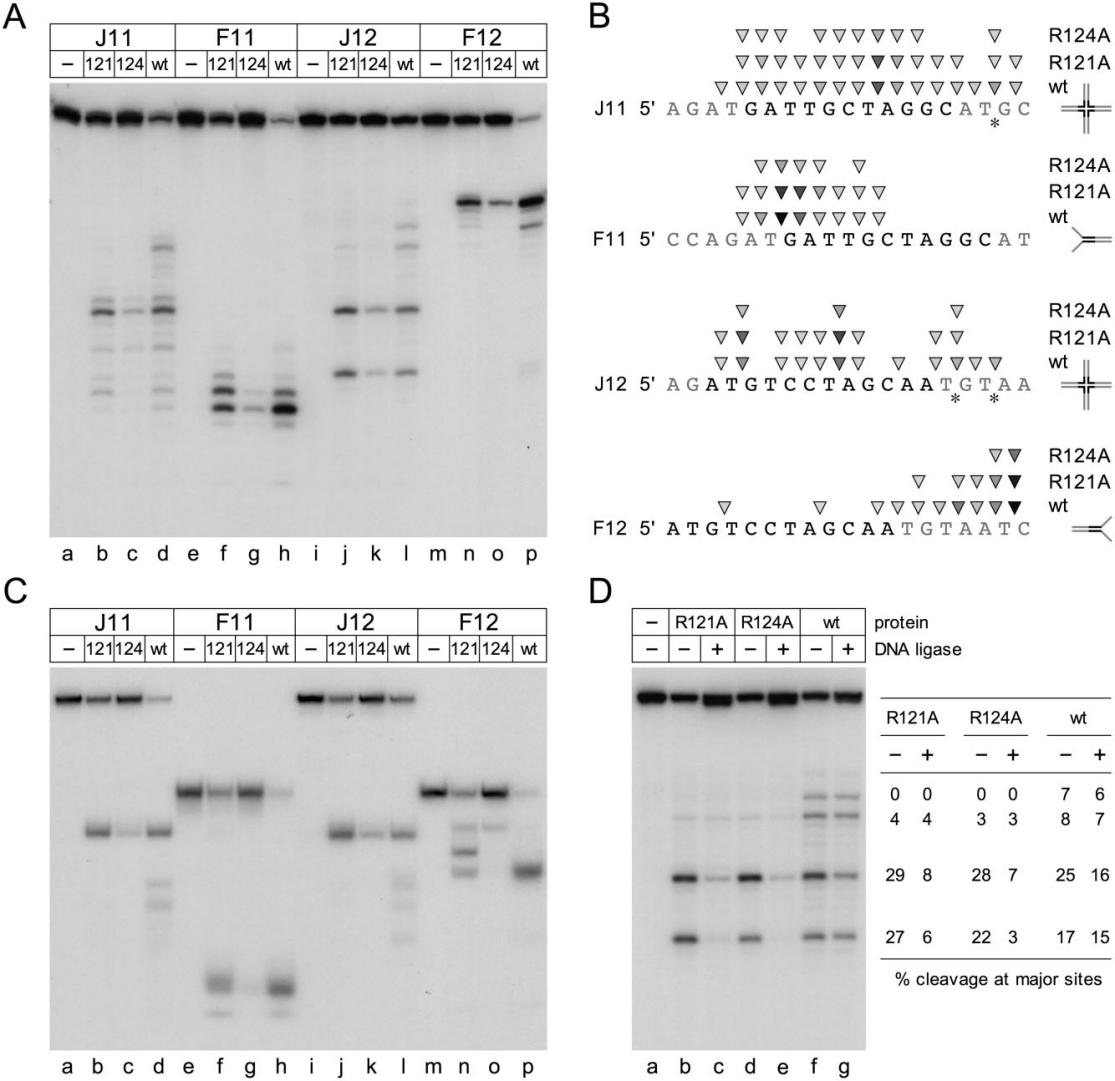
Sequence specificity and symmetry of cleavage by 67RuvC R121A and R124A mutant proteins. A. Mapping of R121A, R124A and wt 67RuvC cleavage sites on junction (J11 and J12) and fork (F11 and F12) DNA substrates. Reactions contained 10 mM MgCl_2_, 0.6 nM ^32^P-labelled DNA and protein at 10 nM. Lanes a, e, i and m served as no protein controls. Reactions were incubated for 15 min at 37°C before separation on 10% denaturing PAGE. B. Location of incisions on the ^32^P-labelled strand of each substrate. The homologous core of junctions J11 and J12 are indicated in black flanked by heterologous sequences in grey; in the F11 and F12 fork substrates this core homology is also highlighted despite being fully annealed to its complement at one end. Cut sites were located by comparison with previous mapping data with wt 67RuvC (Curtis *et al*., 2005) and are indicated by triangles, with the intensity of shading proportional to the amount of cleavage at a particular position. Asterisks indicate major asymmetrical incisions made by wt 67RuvC lying outside the region of homology in J11 and J12 (Curtis *et al*., 2005). C. Products of cleavage by R121A, R124A and wt 67RuvC on junction (J11 and J12) and fork (F11 and F12) DNA substrates. Reactions were performed as in (A) with DNA separated on a 10% neutral gel to visualize the products of cleavage. D. Ligation of the products of R121A, R124A and wt 67RuvC Holliday junction resolution. Reactions contained 10 mM MgCl_2_, 0.6 nM ^32^P-labelled J12 and 40 nM protein and were incubated at 37°C for 15 min. ATP (1 mM) was added and half of each reaction transferred to a fresh tube. T4 DNA ligase (2.5 units) was added to one half of each reaction and incubation at 37°C continued for a further 15 min. Samples were separated on a 10% denaturing polyacrylamide gel and analysed by phosphorimaging. ImageJ was used to quantify the amount of ligation at the four major sites cleaved by wt 67RuvC. Values are expressed as a percentage of the total radioactivity units detected in each lane in the presence (+) or absence (−) of DNA ligase.

Modifications in the nucleotide sequence specificity of cleavage by R121A and R124A could explain why these mutants exhibit reduced proficiency in DNA branch resolution. To uncover any change in preferred cleavage consensus, the location of incisions on a single strand of the J11 and F11 junctions was determined (Fig. 7). The cut sites of a related Holliday junction substrate with a 12 bp homologous core (J12) and another fork derived from this (F12) were also mapped to ensure that a range of potential sites were monitored. The products of DNA branch resolution were analysed by denaturing (Fig. 7A) and neutral (Fig. 7C) gel electrophoresis to identify cut sites and visualize the differing products of resolution. Comparing the results with previous studies with wt 67RuvC (Curtis *et al*., 2005) allowed any alterations in symmetrical and asymmetrical positions to be assessed.

Mapping the cut sites on the two fork junctions revealed minor changes in the level of cleavage at particular sites close to the branch point, for example with R121A on F11 and F12 relative to wt 67RuvC (Fig. 7A, lanes f and h, n and p). This suggests that R121 does play some role, either directly or indirectly, in influencing the location of incisions at preferred sites. No changes in nucleotide sequence selectivity were evident with R124A. Interestingly, while the products of resolution with all three proteins on F11 were as expected when analysed on neutral gels (Fig. 7C, lanes f–h), the two mutants showed a different banding pattern compared with the wt on F12 (Fig. 7C lanes n–p). These probably represent intermediate degradation products that have lost a single tail of the fork, rather than both, as appears to be the case with the wt. This could be a consequence of decreased activity, reduced binding to forks that have already lost one strand or subtle alterations in sequence specificity that influence the types of products that can be generated.

In J11 and J12, wt 67RuvC cuts the junction close to the mobile cross-over point with major sites on the 3′ side of thymidine residues (Fig. 7A and B; Curtis *et al*., 2005). A similar pattern of incisions was obtained with R121A and R124A, although the latter protein shows considerably less activity (Fig. 7A and B). The most striking difference between mutants and wt was the reduction in incisions that lie in heterologous segments outside of the mobile junction core at the 5′-AT↓GC-3′ site in J11 and overlapping 5′-AT↓GT-3′ and 5′-GT↓AA-3′ sites in J12 (Fig. 7B, marked by asterisks). The relative absence of nicking at these sites with R121A and R124A explains why the additional junction breakdown products seen with the wt (Fig. 5E, lanes b–d and Fig. 7C, lanes d and l) are not observed with the mutant proteins (Fig. 7C, lanes b–c and j–k). Thus the mutants not only show enhanced Holliday junction binding selectivity, they display enhanced resolution specificity that favours symmetry-related incisions at matching sequences to preferentially generate nicked duplex products.

### Resolution symmetry of 67RuvC R121A and R124A mutants on Holliday junction DNA

To confirm that the mutant proteins possess an enhanced symmetry of Holliday junction resolution, the junction cleavage activity on J12 was assayed in the presence or absence of DNA ligase. If the products of resolution are the result of symmetrically related incisions across the branch point, the resulting nicked duplexes will possess 5′-phosphate and 3′-hydroxyl groups suitable for repair by DNA ligase. Cleavage sites that are unrelated by symmetry will yield flaps and truncated duplexes that cannot be restored by ligation. Increased amounts of 67RuvC proteins were used to improve the visualization of R124A cut sites. Following cleavage, T4 DNA ligase was added and the products analysed by denaturing PAGE (Fig. 7D). As noted previously (Curtis *et al*., 2005), the wt produces four major cut sites, the two faster-migrating bands corresponding to sites within the homologous core. These bands decrease in intensity upon addition of ligase with a concomitant increase in the quantity of the full-length oligonucleotide as a consequence of ligation of the nicked DNA (Fig. 7D, lanes f–g). The slower-migrating bands represent incisions outside the region of homology, lack corresponding sites in the opposing strand and therefore cannot be ligated. Similar results were obtained at these positions with the mutant proteins, which cut poorly at these sequences (Fig. 7D, lanes b–e). The two major incisions made by R121A and R124A within the homologous core clearly yield nicked duplexes as the majority of the cut strand in both cases was repaired by DNA ligase (Fig. 7D). Since much less of the DNA was ligated with the wt, even at major sites within the mobile core, it appears that 67RuvC normally makes many single breaks that do not have a symmetrically related partner. In contrast, the mutant proteins cleave with a high degree of symmetry to yield nicked duplex products, a characteristic feature of classical, bacterial Holliday junction resolvases.

## Discussion

### Structural differences between 67RuvC and *Ec*RuvC

The crystal structures of 67RuvC, *Ec*RuvC (Ariyoshi *et al*., 1994) and *Tth*RuvC (Chen *et al*., 2013) reveal equivalent topologies and an analogous dimeric arrangement, consistent with predictions based on sequence homology (Bidnenko *et al*., 1998; Curtis *et al*., 2005). However, the phage and bacterial RuvC dimers vary in the relative conformations adopted by equivalent secondary structure elements and also in the length and organization of intervening loops. As a consequence of these alterations, 67RuvC lacks the pronounced central ridge evident in the *Ec*RuvC dimer, presenting a somewhat flatter appearance to its junction binding interface and slightly narrower DNA binding clefts. These differences arise primarily from the conformations of the loops between β3/αA and β4/αB, along with additional residues found in the loop between β1/β2 and at the N-terminus of αD, which includes R121 and R124. The region at the N-terminal end of αD has noticeably more positively charged residues than the equivalent region in *Ec*RuvC (Ariyoshi *et al*., 1994) or *Tth*RuvC (Chen *et al*., 2013), which could account for the enhanced capacity of 67RuvC to bind a range of DNA substrates compared with the more restricted 4-way Holliday junction selectivity observed with *Ec*RuvC (Benson and West, 1994; Takahagi *et al*., 1994).

### Effects of alanine substitutions on the structure of 67RuvC

Of the 10 single amino acid substitutions introduced in the 67*ruvC* gene, only five conferred a functional defect in screens to monitor replication fork and Holliday junction cleavage *in vivo*. Despite being highly conserved in the phage RuvC family, mutation of residues S10, K40, S109, K110 and K120 to alanine had no significant effect upon activity, although the assays may not be sufficiently sensitive to detect more modest reductions in enzyme catalysis. Examination of the location of these residues in relation to a Holliday junction modelled on the 67RuvC structure, suggests that they may be either too remote from the DNA to be critical (S10, K40, S109 and K110) or are unnecessary for stabilizing the protein–junction complex (K120). The latter instance is perhaps the most surprising but may reflect the large number of positively charged residues located at the N-terminus of αD that could compensate for its absence. In contrast, alanine substitutions at residues T11, R46, R121, R124 and K125 had severe deleterious effects upon fork and Holliday junction cleavage activities *in vivo*, although R121A can partially restore UV resistance in resolvase-deficient *ruvABC* mutants. Residues T11 and R46 are rather remote from the DNA in the 67RuvC–Holliday junction model but lie close to active site residues at the base of the cleft and interact with each other. Thus substitution of either residue by alanine could affect the shape of the cleft and/or the conformation of catalytically important residues nearby. R121, R124 and K125 lie proximal to the DNA in our model where they are inserted into the major groove at the N-terminal end of αD and directly above the active site residues and bound metal cations. Thus they are ideally positioned to play a critical role in DNA substrate recognition. There are no structural equivalents of 67RuvC R121 or R124 in the *Ec*RuvC or *Tth*RuvC structures, although they are conserved among phage RuvC resolvases. However, K125 does superimpose fairly well with K118 in *Ec*RuvC and K122 in *Tth*RuvC and could fulfil a similar function in stabilizing interactions with the phosphodiester backbone linked to catalysis. In *Ec*RuvC, K118 substitution mutations produce defects in Holliday junction binding and resolution (Ichiyanagi *et al*., 1998; Yoshikawa *et al*., 2000). An additional loop (A12-G16) present in 67RuvC, but absent from the bacterial enzymes, incorporates two positively charged lysines (K14 and K15; Fig. 3D). Although this loop is also missing from many phage RuvC orthologues (Fig. 2A), it may serve to provide further contacts to bind and guide the course of the DNA phosphate backbone and again increase DNA affinity. Similarly, when compared with the two bacterial enzymes, there are a larger number of positively charged residues in the loop preceding αB in 67RuvC, which is close to the centre of bound DNA. Thus the phage enzymes seem to have evolved a greater positive surface charge density and in particular an extended, positively charged N-terminus of αD in order to enhance the binding of simple substrates such as forks in addition to Holliday junctions.

### Branched DNA binding and resolution by 67RuvC R121A and R124A mutants *in vitro*

To help clarify the importance of the αD region in DNA structure selectivity, we attempted to make chimeric forms of 67RuvC and *Ec*RuvC (see Fig. S6). Hybrid recombinases have successfully confirmed functionality of related gene segments in T4 endonucleases V and VII (Giraud-Panis *et al*., 1995). Reciprocal exchanges of C-terminal segments located immediately after the dimer interface helix αB allowed the construction of 67-*Ec*RuvC and *Ec*-67RuvC hybrids. Unfortunately, although stable protein hybrids of both were obtained they failed to produce enzymes that displayed endonuclease activity in Holliday junction and fork cleavage assays *in vivo*. Instead we selected mutants in two basic residues at the apex of αD (R121A and R124A) to characterize their impact on binding and resolution of model branched DNA substrates *in vitro*. The R121A mutant displayed partial Holliday junction resolution activity but no replication fork cleaving ability as judged by *in vivo* assays, while R124A was defective in both activities. Significantly, both of the purified mutant proteins displayed an enhanced selectivity for binding to Holliday junction and Y-shaped fork DNA over forks with one or two ssDNA arms and linear duplex substrates. The data suggest that these positively charged residues normally help stabilize interactions more generally with the DNA backbone and this imparts a reduced Holliday junction specificity in phage bIL67 RuvC relative to its bacterial congeners. The effect of these mutant proteins on the resolution of branched structures was also analysed. Both R121A and R124A showed reduced endonuclease activity at sites located in the heterologous arms of Holliday junction substrates containing either an 11 or 12 bp core of homology. Ligation of the products of resolution in the latter substrate revealed that the mutant proteins showed significantly improved resolution symmetry compared with the wt at two major cut sites at the junction cross-over. Thus the mutations confer improved symmetrical resolution of Holliday junctions, a feature more typical of bacterial RuvC and yeast Ydc2 resolvases (Oram *et al*., 1998; Sharples, 2001; Chen *et al*., 2013). Replacement by alanine of either of these positively charged residues in αD must somehow encourage a more optimal positioning of scissile phosphates for dual strand incision.

The R121A and R124A mutants retained some endonuclease activity on fork junctions *in vitro*, although not *in vivo*, confirming that these branched substrates, although bound less well, remain a target. Hence additional elements within the phage RuvC secondary structure are likely to contribute to resolution specificity, potentially by partially uncoupling the ‘nick-counter-nick’ mechanism of resolution employed by bacterial RuvC proteins that ensures dual strand scission across the junction cross-over (Fogg and Lilley, 2000; Chen *et al*., 2013).

An ability to target a range of structures, in addition to Holliday junctions, confers an advantage on phage RuvC resolving enzymes by facilitating the removal of any branched molecules that could potentially interfere with genome packaging. A similar role is fulfilled by the resolving enzymes from bacteriophages T4 and T7 (Kemper and Brown, 1976; Tsujimoto and Ogawa, 1978). The ability to cleave bacterial replication forks may also serve to liberate nucleotides through chromosome degradation that can be incorporated in viral DNA synthesis (Center *et al*., 1970). The work presented here sheds light on the architectural variation in RuvC family proteins required to distinguish and cleave different branched DNA structures, features that are likely to be conserved in other unrelated resolving enzymes. Significantly, from an evolutionary perspective, it reveals that relatively few modifications are apparently necessary to convert a Holliday junction specific endonuclease that resolves by paired symmetry-related incisions into a more versatile debranching endonuclease that is suited to the particular requirements of phage DNA metabolism.

## Experimental procedures

### Phage bIL67 RuvC mutant constructs

An *E. coli* codon-optimized version (Fig. S7) of the *Lactococcus lactis* phage bIL67 *ruvC* (*ORF23*) gene (Schouler *et al*., 1994) was synthesized and inserted into the T7 expression vector pET24a(+) at NdeI and BamHI sites by GenScript to create p67RuvCwt. Site-directed mutant derivatives of this clone were also generated by the company at the same time. A K110A mutant (pFC254) was made by site-directed mutagenesis using oligonucleotides 5′-ATTGATAACTCAGCATGGTGTAGCT-3′ and 5′-AGCTACACCATGCTGAGTTATCAAT-3′ with pFC105 carrying the 67*ruvC* wt gene in pET24a (Curtis *et al*., 2005). All constructs were verified by nucleotide sequencing.

### Holliday junction resolution *in vivo*

The *E. coli* K-12, resolution-defective, Δ*ruvABC*::*cat* mutant (N4454) is a derivative of the *ruvABC*^+^ wt strain, AB1157 (Curtis *et al*., 2005). The ultraviolet light sensitivity of these strains harbouring wt and mutant versions of bIL67 *ruvC* was determined by growing transformed cells in LB broth at 37°C to an A_650nm_ of 0.4 and spotting serial 10-fold dilutions onto LB agar plates containing 40 μg ml^−1^ kanamycin. Plates were exposed to UV light at a dose rate of 1 J m^2^ s^−1^, incubated for 17–21 h at 30°C and the fraction surviving calculated with reference to an unirradiated control.

### Replication fork cleavage *in vivo*

*Escherichia coli* BL21-AI strains were employed as a host for expression of wt and mutant 67RuvC proteins. Bacteria (8 ml) were grown to an A_650nm_ of 0.6 in LB broth containing 40 μg ml^−1^ kanamycin at 37°C and gene expression induced in half of the culture by addition of IPTG (1 mM) and arabinose (0.2%). Incubation was continued for 1 h before uninduced and induced cells (3 ml) were harvested by centrifugation. Plasmid and chromosomal DNA fragments were isolated by the Qiagen miniprep protocol and analysed by 1% agarose gel electrophoresis. Molecular weight markers were 100 bp and 1 kb ladders from New England Biolabs. The remaining 1 ml of cells from each uninduced and induced culture was harvested and subjected to 12.5% SDS-PAGE to monitor 67RuvC protein expression. Precision Plus Protein™ Kaleidoscope Standards (Bio-Rad) were used as a marker and gels were stained with Coomassie blue.

### Proteins

67RuvC wt protein was purified as described (Curtis *et al*., 2005). 67RuvC R121A and R124A were purified from 500 ml of *E. coli* BL21-AI carrying the appropriate plasmid construct. Cells were grown to A_650nm_ 0.5 in LB containing kanamycin (40 μg ml^−1^) and expression induced with 1 mM IPTG and 0.2% arabinose for 3 h at 37°C. Harvested cells were resuspended in Buffer A (20 mM Tris-HCl pH 7, 1 mM EDTA, 0.5 mM DTT) and lysed by sonication. The clarified lysate was applied to a 3 ml cellulose phosphate (Sigma) column in Buffer A and bound proteins eluted with a linear gradient of 0–1 M KCl. The majority of the 67RuvC mutant protein eluted between 0.5 and 0.8 M KCl and pooled fractions were dialysed overnight in Buffer A. As with the purification of 67RuvC D8N (Curtis *et al*., 2005), this induced the majority of the phage RuvC protein to precipitate. Centrifugation at 2300 *g* for 10 min yielded a protein pellet, which was resolubilized in Buffer A containing 0.6 M KCl. Both mutant proteins retained some minor contaminating proteins and R121A was further purified (> 95% pure) by gel filtration on a 24 ml Superose 12 HR 10/30 column (GE Healthcare) in Buffer A containing 0.5 M KCl. Aliquots of R121A and R124A were stored at −80°C in Buffer A pH 7 containing 0.5 M KCl and 50% glycerol.

Protein concentrations were determined in a NanoDrop 2000 (Thermo Scientific) micro-volume spectrophotometer; amounts of protein are expressed as moles of dimeric protein. Restriction endonucleases, T4 DNA ligase, T4 polynucleotide kinase and Pfx DNA polymerase were purchased from Invitrogen.

### X-ray crystallography

The 67RuvC D8N mutated variant in a pET24a(+) vector containing a kanamycin resistance gene marker was created as described previously (Curtis *et al*., 2005) and transformed cells grown and harvested as above. Protein was purified using a heparin-sepharose column with a 0.2–0.8 M NaCl gradient in 50 mM Tris-HCl pH 8, followed by a sephadex gel filtration column in 0.5 M NaCl, 50 mM Tris-HCl pH 8 and finally concentrated to 20 mg ml^−1^ using a Vivaspin concentrator unit. Crystallization conditions were screened using commercial kits and successfully optimized. The selenomethionine form of the protein used for structure determination was grown using the same protocols as the native but in minimal media incorporating 40 mg l^−1^ selenomethionine to replace methionine. The protein was purified in an identical manner and the level of selenomethionine incorporation confirmed to be ≥ 90% by mass spectrometry.

Crystals with a rod-like morphology were grown in 20% ^w^/_v_ PEG3350, 0.1 M KCl, 0.1 M Tris-HCl pH 7.5 and reached approximately 0.2 × 0.05 × 0.05 mm after 4 days. X-ray diffraction data were collected on a home laboratory source which extended to 2.3 Å and revealed a unit cell with dimensions of a = 63.1 Å, b = 66.7 Å, c = 74.0 Å and α = β = γ = 90°. A full three wavelength MAD phasing experiment was carried out on station BM14 at the ESRF (Grenoble, France). Processing was carried out using the HKL suite (Otwinowski and Minor, 1997) and the space group identified using XPREP/SHELXD from the SHELX program suite (Sheldrick, 2010), which was also used to locate the heavy atoms and calculate phases for an initial map at 2.5 Å resolution (Table S1). Initial automated building was carried out using ARP/wARP before refinement using REFMAC (Murshudov *et al*., 1997) from the CCP4 suite (Winn *et al*., 2011) interspersed with manual model building using COOT (Emsley and Cowtan, 2004). A high resolution data set was collected to 1.7 Å resolution on the same beamline at an energy of 14000 eV and processed later with MOSFLM (Leslie and Powell, 2007) before being merged and scaled with SCALA (Evans, 2006). It was used to carry out final refinement of the model to 1.8 Å resolution. Validation of the structure was carried out using MOLPROBITY (Chen *et al*., 2010).

A structure was also obtained using native protein but grown from 0.4 M MgCl_2_, 25% ^w^/_v_ PEG2000, 0.1 M HEPES pH 7. The high concentration of Mg^2+^ was used in the hope of compensating for the expected reduced metal affinity arising from the D8N mutation. Data were collected on beamline I02 at Diamond Light Source (near Oxford, UK). The crystal diffracted out to a maximum resolution of 1.8 Å and were initially processed in MOSFLM before being merged and scaled with SCALA. The structure was determined by molecular replacement using PHASER (McCoy *et al*., 2007), and the model adjusted and refined once again using COOT and REFMAC. In the latter stages of refinement, Mg^2+^ ions were modelled into the structure consistent with appropriate co-ordination geometries and ligand distances. Model validation was carried out using MOLPROBITY. Model co-ordinates and structure factors have been deposited at the PDB with accession codes 4KTW (free protein) and 4KTZ (Mg^2+^ bound).

### Construction of DNA substrates

Holliday junction (J11/J12), fork (F11/F12) and duplex (D11/D12) DNA substrates were made by annealing synthetic 49–51 mer oligonucleotides (Table S2). Strands 11-1 and 12-2 were 5′-end labelled with ^32^P using [γ-^32^P] ATP (Perkin-Elmer) and T4 polynucleotide kinase. F11 and F12 fork structures were made by annealing two strands from junction J11 (11-1 and 11-2) and J12 (12-1 and 12-2) respectively. F12-Y and F12-RF were made using 12-1 and 12-2 with addition of the 18–19 mer oligonucleotides 12-6 and 12-7 (F12-Y) or 12-7 (F12-RF); strand 12-1 was ^32^P-5′-end labelled in these two forks. D11 was made by annealing 11-1 to 11-5 and D12 by annealing 12-2 to 12-5. All substrates were gel-purified on 10% PAGE.

### DNA binding and cleavage assays

Band shift assays (20 μl) using ^32^P-labelled DNA substrates were carried out in 50 mM Tris-HCl pH 8.0, 5 mM EDTA, 1 mM dithiothreitol, 5% glycerol, 100 μg ml^−1^ BSA. Samples were incubated on ice for 15 min before separation on 4% PAGE in 6.7 mM Tris-HCl pH 8.0, 3.3 mM sodium acetate, 2 mM EDTA at 10 V cm^−1^ for 1 h 15 min. Cleavage of ^32^P-labelled DNA was assayed at 37°C for 15 min in 50 mM Tris-HCl pH 8.0, 1 mM dithiothreitol, 100 μg ml^−1^ BSA, 10 mM MgCl_2_. Reactions (20 μl) were terminated by the addition of 5 μl of 100 mM Tris-HCl pH 8.0, 2.5% SDS, 100 mM EDTA, 10 units μl^−1^ proteinase K (Sigma) and incubated for a further 10 min at 37°C. Following addition of 5 μl of loading buffer (0.25% w/v bromophenol blue, 0.25% w/v xylene cyanol, 15% v/v Ficoll type 400), 15 μl was electrophoresed on 10% polyacrylamide gels in 90 mM Tris-borate, 2 mM EDTA at 12 V cm^−1^ for 1 h 45 min. To determine the location of cleavage sites, terminated reactions (5 μl) were mixed with 2 μl of 0.3% w/v bromophenol blue, 0.3% w/v xylene cyanol, 10 mM EDTA and 97.5% v/v formamide. Samples were boiled for 2 min prior to electrophoresis on 15% 7 M urea-TBE sequencing gels at 44 V cm^−1^ for 2 h. Gels were dried onto filter paper and visualized by autoradiography and phosphorimaging. Data were analysed using ImageQuant and ImageJ software. Apparent *K_D_* values were determined using GraphPad Prism 4.0 software, fitting the data using a sigmoidal dose–response curve with variable slope.
