# Supplementary material for: Mutants of phage bIL67 RuvC with enhanced Holliday junction binding selectivity and resolution symmetry
Source: Mol Microbiol. 2013 Aug 14;89(6):1240–58. doi: 10.1111/mmi.12343 (PMC3864405; doi:10.1111/mmi.12343)
Supplement: Supplementary file 1 [file mmi0089-1240-SD1.pdf]

Supporting information

Mutants of phage bIL67 RuvC with enhanced Holliday junction binding selectivity and resolution symmetry

Victoria Green, Fiona, A. Curtis, Svetlana Sedelnikova, John B. Rafferty and Gary J. Sharples

Table S1. Data statistics from MAD phasing experiment.

Data set	Peak	Inflection	Rem
Wavelength eV (Å)	12670.55 (0.9785)	12668.19 (0.9787)	14000.02 (0.88560)
Resolution (Å)	50.00-2.45	50.00-2.45	50.00-2.22
(Highest shell)	(2.54-2.45)	(2.54-2.45)	(2.30-2.22)
Unique Reflections	11901	11908	15922
Multiplicity	7.8 (7.7)	7.8 (7.7)	7.9 (7.3)
Completeness %	99.2 (96.9)	99.5 (99.6)	99.7 (99.8)
Mean (I)/σ(I)	56.93 (28.1)	55.78 (30.47)	49.8 (17.6)
R_{merge}	0.049 (0.094)	0.046 (0.096)	0.047 (0.169)
f"/f'	5.46/-7.82	3.37/-9.80	3.15/-1.28

MAD data statistics from the peak, inflection and high energy remote (Rem) data. Values in parentheses are for the highest resolution shells.

Crystals were obtained from a precipitant mixture of 20% w/v PEG3350, 0.1 M KCl and 0.1 M Tris-HCl pH 7.5. They grew with a rod-like morphology to approximately 0.2 x 0.05 x 0.05 mm after 4 days. X-ray diffraction data were collected initially on a home laboratory source and extended to 2.3 Å, revealing a unit cell with dimensions of a=63.1 Å, b=66.7 Å, c=74.0 Å and $\alpha=\beta=\gamma=90°$ in a P-orthorhombic space group subsequently confirmed as $P2_12_12_1$.

The heavy atom location carried out using SHELXD from the HKL2MAP suite of programs found 3 of the 4 potential sites in the asymmetric unit with a Patterson figure of merit of 57.1. Maps using both enantiomeric forms were generated at 2.5 Å resolution and submitted to density modification in SHELXE. The correct form gave overall contrast and connectivity values of 0.44 and 0.90, respectively and a pseudo-free correlation coefficient of 65% versus 18% for the incorrect hand.

Table S2. Oligonucleotides used in the construction of DNA substrates.

Oligo	Sequence (5'-3')
11-1	GGCGACGTGATCACCAGATGATTGCTAGGCATGCTTTCCGCAAGAGAAGC
11-2	GGCTTCTCTTGCGGAAAGCATGCCTAGCAATCCTGTCAGCTGCATGGAAC
11-3	GGTTCCATGCAGCTGACAGGATTGCTAGGCTCAAGGCGAACTGCTAACGG
11-4	ACCGTTAGCAGTTCGCCTTGAGCCTAGCAATCATCTGGTGATCACGTCGC
11-5	GCTTCTCTTGCGGAAAGCATGCCTAGCAATCATCTGGTGATCACGTCGCC
12-1	GACGCTGCCGAATTCTGGCTTGCTAGGACATCTTTGCCCACGTTGACCC
12-2	TGGGTCAACGTGGGCAAAGATGTCCTAGCAATGTAATCGTCTATGACGTT
12-3	CAACGTCATAGACGATTACATTGCTAGGACATGCTGTCTAGAGACTATCGA
12-4	ATCGATAGTCTCTAGACAGCATGTCCTAGCAAGCCAGAATTCGGCAGCGT
12-5	AACGTCATAGACGATTACATTGCTAGGACATCTTTGCCCACGTTGACCCA
12-6	GCCAGAATTCGGCAGCGT
12-7	AACGTCATAGACGATTACA

Mobile core regions in J11 and J12 are underlined.

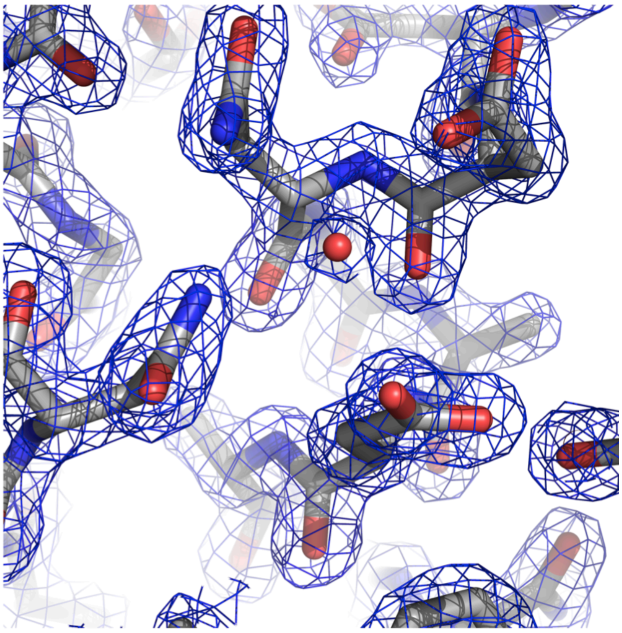

Figure S1

Fig. S1. A section of the σA-weighted 2mFo-DFc map shown as blue mesh contoured at 1.5σ in the region of the catalytically critical residues. The protein has been shown in stick representation and the solvent molecules as spheres (carbon grey; oxygen red; nitrogen blue). Produced using PyMOL.

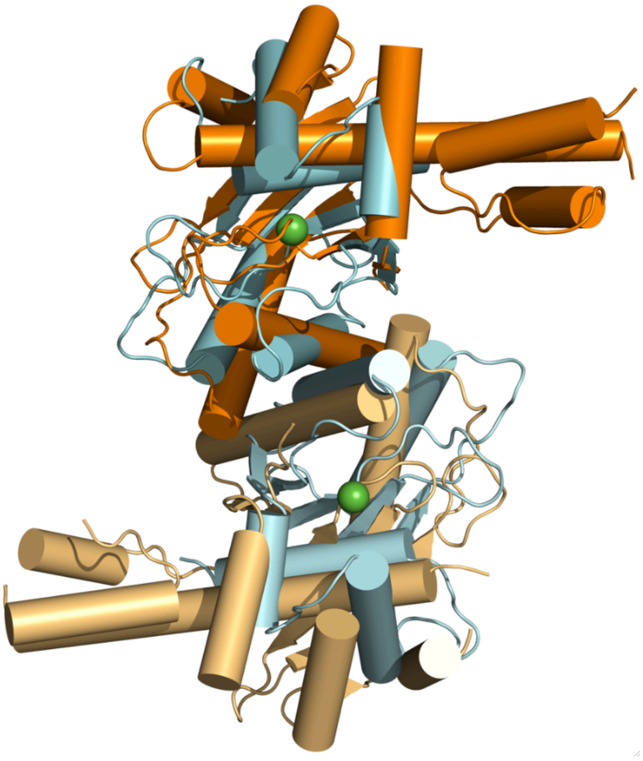

Figure S2

Fig. S2 (previous page)**.** Superimposition of a dimer of 67RuvC with that of Ydc2. Structures are viewed along the dimer two-fold axis of 67RuvC and shown in cartoon representation (67RuvC monomers coloured cyan and aquamarine; Ydc2 monomers coloured orange and light orange). The superimposition was *via* the β-sheet of the upper monomer in each dimer. Note the extra helical domains in Ydc2 at upper right and bottom left of the image. The bound Mg^{2+} cations (green spheres) at the active sites of 67RuvC are shown for reference.

Whilst Ydc2 possesses the RNase H fold present in other integrases and still forms a dimer, it also has an extra domain, formed by a helical bundle, to which there is no equivalent in the bacterial or phage RuvC enzymes. The corresponding helices and intervening loops in Ydc2 are also generally longer and have noticeably different relative locations to the central mixed β-sheet. The Ydc2 dimer interface is similar to that of 67RuvC in that it is dominated by a single helix from each monomer but these are less parallel than seen in 67RuvC and *Ec*RuvC and there are fewer hydrogen bonds formed. Each active site of Ydc2 consists of a cluster of five acidic residues (D46, E117, D226, D227 and D230) believed to bind two Mg^{2+} cations during catalysis. The separation distance of the active sites in Ydc2 is greater than in 67RuvC in keeping with the differences in dimer interfaces, which leads to poor superimposition of the second monomer in each dimer after superimposition of the first. However, when a single active site from each monomer is considered, there is greater correspondence between 67RuvC and Ydc2 than is seen for 67RuvC and *Ec*RuvC. Residues D46, E117, D226, D227 and D230 in Ydc2 can be seen to superimpose with residues N8 (D8 in wt), E71, D145, N146 and D149 in 67RuvC (Fig. 2C). In 67RuvC, N146 has not previously been associated with cation binding, although the nearest residue to it in a structure alignment with *Tth*RuvC is the H143 residue, which is acknowledged as one of the important active site residues. In addition, nearby N51 in Ydc2 corresponds structurally with E17 in 67RuvC for which there is no equivalent in *Ec*RuvC.

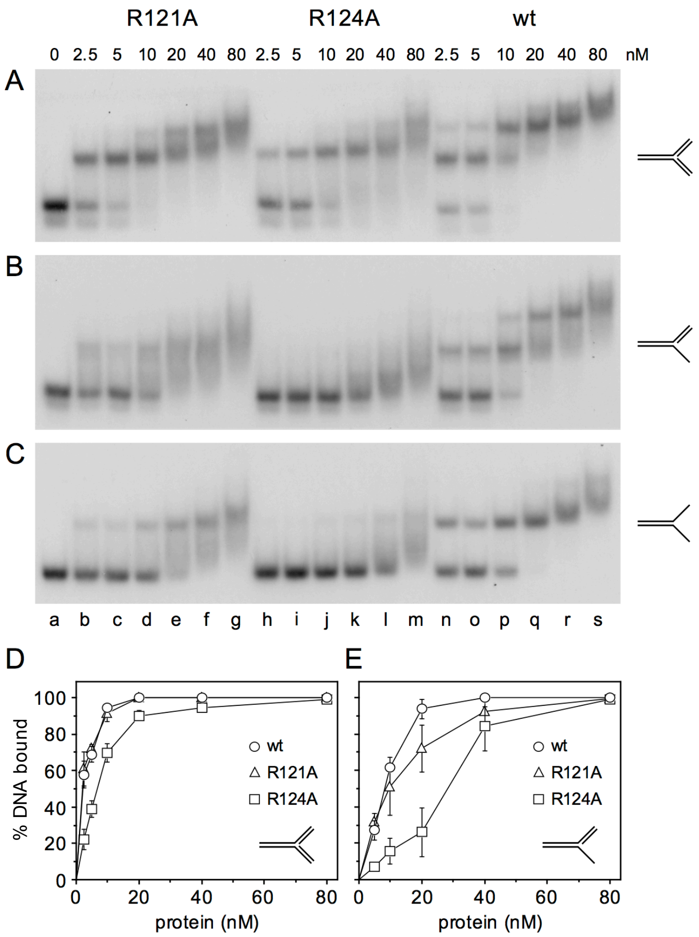
R121A
R124A
wt
0 2.5 5 10 20 40 80 2.5 5 10 20 40 80 2.5 5 10 20 40 80 nM
A
B
C
a b c d e f g h i j k l m n o p q r s
D
E
% DNA bound
protein (nM)
wt
R121A
R124A

Figure S3

Fig. S3 (previous page)**.** Fork DNA binding by 67RuvC R121A and R124A mutant proteins. (A-C) Gel retardation assays showing binding of R121A (lanes b-g), R124A (lanes h-m) and wt 67RuvC (lanes n-s) to F12-Y (A), F12-RF (B) and F12 (C) DNA substrates. Lane a served as no protein control. Binding reactions contained 5 mM EDTA, 0.15 nM ^{32}P-labelled DNA and protein at 2.5, 5, 10, 20, 40 and 80 nM. Samples were incubated on ice for 15 minutes before separation on 4% PAGE. (D-E) Percentage binding by 67RuvC R121A, R124A and wt proteins on F12-Y (D) and F12-RF (E). Data are the mean and standard deviation of two independent experiments. Significant smearing of F12-RF with R121A and R124A proteins made quantification of bound DNA more difficult to evaluate, explaining the larger error bars in these two cases.

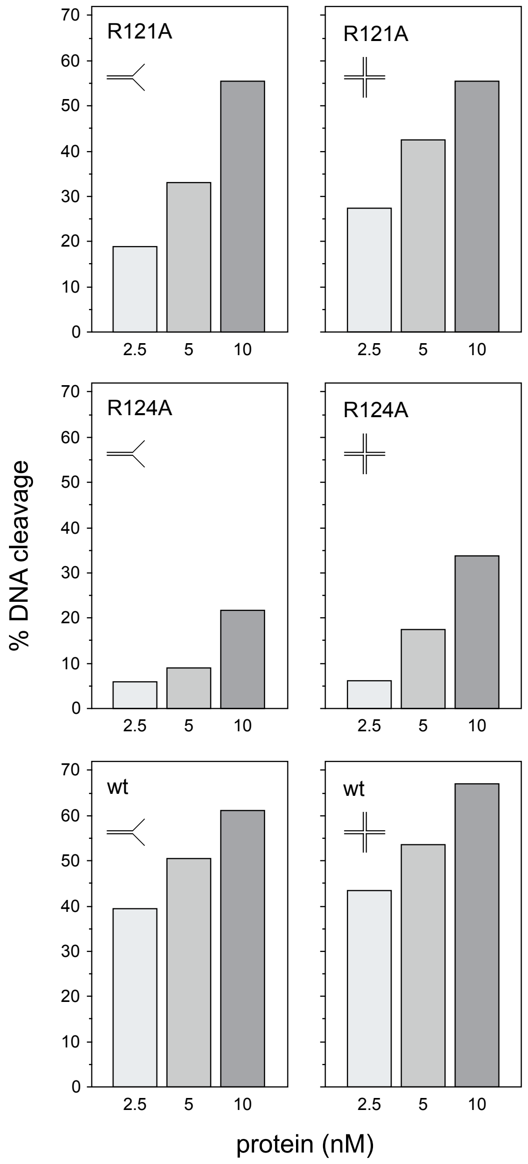
R121A
R124A
wt
% DNA cleavage
protein (nM)
2.5
5
10
0
10
20
30
40
50
60
70

Figure S4

Fig. S4 (previous page)**.** Analysis of fork and Holliday junction DNA cleavage activities of 67RuvC mutant proteins. Percentage cleavage by 67RuvC R121A, R124A and wt proteins on F11 and J11 are shown. Data were quantified by phosphorimaging from the gels presented in Figure 5D-F.

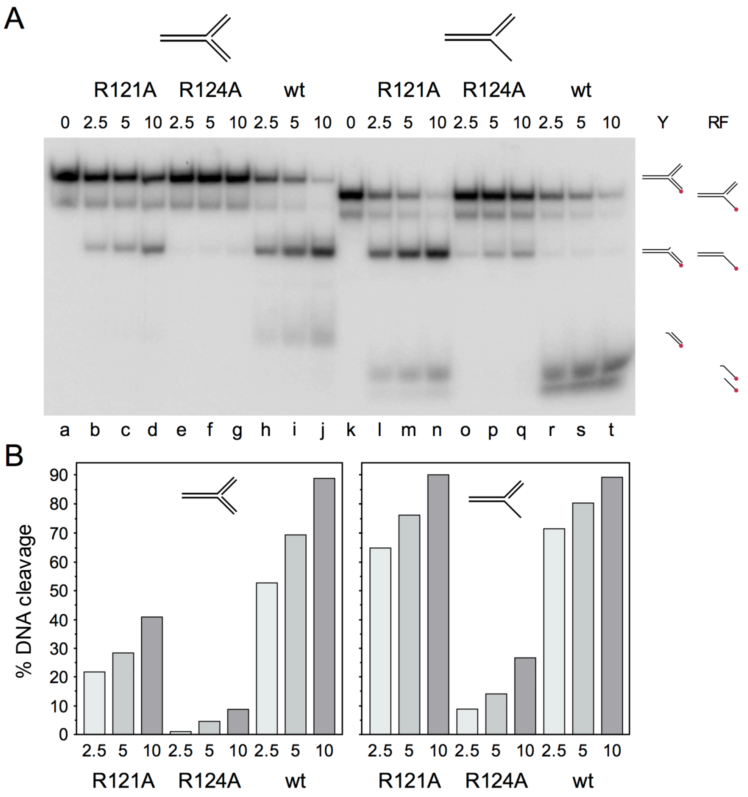
A
R121A R124A wt R121A R124A wt
0 2.5 5 10 2.5 5 10 2.5 5 10 0 2.5 5 10 2.5 5 10 2.5 5 10
Y RF
a b c d e f g h i j k l m n o p q r s t
B
% DNA cleavage
90 80 70 60 50 40 30 20 10 0
2.5 5 10 2.5 5 10 2.5 5 10
R121A R124A wt
2.5 5 10 2.5 5 10 2.5 5 10
R121A R124A wt

Figure S5

Fig. S5 (previous page). Fork DNA cleavage by 67RuvC R121A and R124A mutant proteins. (A) DNA branch cleavage assay showing the resolution products of R121A, R124A and wt 67RuvC on F12-Y (lanes a-j) and F12-RF (lanes k-t) DNA substrates. Reactions contained 10 mM $MgCl_2$, 0.15 nM ^{32}P-labelled DNA and protein at 2.5, 5 and 10 nM; R121A (lanes b-d and l-n), R124A (lanes e-g and o-q) and wt (lanes h-j and r-t). Lanes a and k served as no protein controls. Reactions were incubated for 15 minutes at 37°C before processing and separation on 10% PAGE. Reaction substrates and products are indicated on the right with a red circle indicating the position of the ^{32}P label. Both F12-Y and F12-RF substrates are less stable than F11 under reaction conditions with the loss of the shorter oligonucleotides resulting in the appearance of a small quantity of slightly faster migrating fork species even in the absence of protein. (B) Percentage cleavage by 67RuvC R121A, R124A and wt proteins on F12-Y and F12-RF. Data were quantified by phosphorimaging from the gel presented. Although R121A and wt show similar percentages of DNA cleaved, it should be noted that the reaction has proceeded much further with the latter with most of the labelled DNA appearing as short ssDNA products.

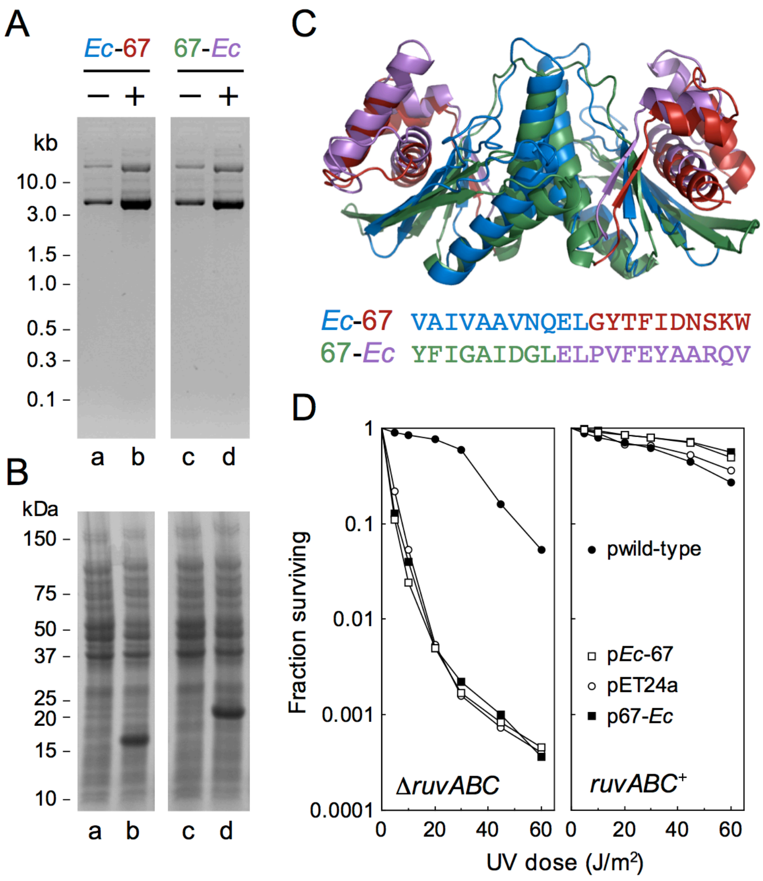
A
Ec-67
67-Ec
kb
10.0
3.0
1.5
1.0
0.5
0.3
0.1
a b c d
B
kDa
150
75
50
37
25
20
15
10
a b c d
C
Ec-67 VAIVAAVNQELGYTFIDNSKW
67-Ec YFIGAIDGLELPVFEYAARQV
D
Fraction surviving
1
0.1
0.01
0.001
0.0001
ΔruvABC
ruvABC+
pwild-type
pEc-67
pET24a
p67-Ec
0 20 40 60
UV dose (J/m2)

Figure S6

Fig. S6 (previous page). Analysis of phage and bacterial RuvC chimeras. (A) Effect on fork cleavage *in vivo*. BL21-AI strains carrying the *Ec*-67 and 67-*Ec* RuvC hybrid constructs were grown to an A_{650nm} of 0.6. Uninduced (–) and arabinose/IPTG-induced (+) cells were harvested and plasmid DNA isolated prior to separation on a 1% agarose gel and staining with ethidium bromide. (B) Analysis of protein expression. Uninduced and induced cells were prepared as in (A) with total cell proteins analysed by 12.5% SDS-PAGE. Products from each matched well to the predicted molecular mass for *Ec*-67RuvC of 17.3 kDa (157 amino acids) and 67-*Ec*RuvC of 19.6 kDa (177 amino acids). (C) Comparison of bacterial and phage RuvC structures highlighting the swapped regions in each hybrid. The aligned sequences show where the hybrid junctions were inserted between αB and β5. (D) Effect on Holliday junction resolution *in vivo*. Relevant genotypes are indicated within each panel and the UV light survival of selected mutants shown alongside wild-type 67RuvC and pET24a(+) control plasmids.

EcRuvC-67RuvC chimeras

In an alternative approach to investigate the importance of αC-αD region for DNA branch specificity, *Ec*-67 and 67-*Ec* RuvC hybrid genes were constructed in the pET24a(+) expression vector. The N-terminal half of *Ec*RuvC up to αB (1-97) was fused to the C-terminus of 67RuvC from β5 (102-161) to give an *Ec*-67RuvC hybrid (Fig. S6C). The reciprocal chimera was generated by joining phage (1-99) and bacterial (98-172) RuvC segments to give 67-*Ec* (Fig. S6C). In both cases potential structural perturbations were minimized by positioning the handover within a poorly conserved loop between αB and βE (Fig. 2A in main text). The *Ec*-67 and 67-*Ec* chimeric *ruvC* genes were made from pFC105 by combining PCR products with an engineered SacI site placed at the appropriate internal site. The product pairs were amplified using *Ec*RuvC-N (5'-GAGACGCCATATGGCTATTATTCTC-3' and 5'-CAAA TACTGGGAGCTCCTGATTCAC-3'), *Ec*RuvC-C (5'-GTGAATCAGGAGCTCCCAGTA TTTG-3' and 5'-CTGACCGAAGCTTTATAACTTAACG-3'), 67RuvC-N (5'-GGATACA TATGAAGAAAATTTTAGC-3' and 5'-AAGTATAGCCGAGCTCTAGACCGTC-3'), 67RuvC-C (5'-TTGACGGTCTAGAGCTCGGCTATAC-3' and 5'-TATAAGCTTACAAT TAACCCAAGTG-3'). NdeI-SacI PCR products were first inserted into pET24a(+), followed by the SacI-HindIII inserts to yield pFC220 (*Ec*-67) and pFC222 (67-*Ec*). The hybrid constructs were introduced into BL21-AI and found to express products of

the predicted molecular mass after addition of IPTG and arabinose (Fig. S6B). However, neither hybrid showed activity in replication fork nicking (Fig. S6A) or Holliday junction resolution (Fig. S6D) assays *in vivo*. It is likely that the 67RuvC and *Ec*RuvC proteins vary sufficiently to prevent proper alignment of catalytic residues thus blocking endonuclease functionality.

```
                         S10A GCTGCC T11A
 NdeI               G           C        C                 C  T         C T
CATATGAAGAAAATTTTAGCTATTGATTTCAGTACCGCTAGTAAGAAAGGCGAGGGTACAGGGTACGCTTTTAGA
   M  K  K  I  L  A  I  D  F  S  T  A  S  K  K  G  E  G  T  G  Y  A  F  R
                              A  A

                                        K40A GCA          R46A GCT
                          G                 G
AAAGACGGTCAAGTTTATGTTGGTTCTATTAAAGCATATAACCCTAAAAAGACCGCTTGGGAACGTACCTTTGAC
K  D  G  Q  V  Y  V  G  S  I  K  A  Y  N  P  K  K  T  A  W  E  R  T  F  D
                                             A                 A

                                                        G           G  G
ATCGTAAACGCAATTAAAGATATTATTGATGAGTTTGATTTGAAAGGTTATCATCTAGCCATTGAAACTCCTATT
I  V  N  A  I  K  D  I  I  D  E  F  D  L  K  G  Y  H  L  A  I  E  T  P  I

      C T   C C        C     C                                            T
ATGGGAAGAAACAGAAAACACAGTATTACATTGGCTAATTGTAACGGTTATTTTATCGGTGCTATTGACGGTCTA
M  G  R  N  R  K  H  S  I  T  L  A  N  C  N  G  Y  F  I  G  A  I  D  G  L

                                                                  R124A GCC
                     S109A GCA                        K120A GCAGCA R121A
              C                                                  T
GTAAATGGCTATACTTTTATTGATAACTCAAAATGGTGTAGCTATCATCTTATTTCAGGCAAACGAGAACAACGC
V  N  G  Y  T  F  I  D  N  S  K  W  C  S  Y  H  L  I  S  G  K  R  E  Q  R
                           A                                A  A        A

GCA K125A
           C                 A  G  T           G
AAAGAAGAAAGTCTTGAACTTTTAAAAGCCACAGGCTTGGTTGATTCTAATTGCAAAGATGACAACATCGCAGAC
K  E  E  S  L  E  L  L  K  A  T  G  L  V  D  S  N  C  K  D  D  N  I  A  D
A

                 C                      BamHI
GCTTACAACATCTTGACATATTGTGAACACTTGGGTTAAGGATCC
A  Y  N  I  L  T  Y  C  E  H  L  G  *
```

Figure S7

Fig. S7 (previous page)**.** Codon optimization and site-directed mutagenesis of the bIL67 *ruvC* gene. Changes to the wild-type bIL67 *ruvC* gene are indicated above the nucleotide sequence in black. Site-directed mutants are highlighted in red. Restriction sites used in cloning are shown in blue.
